# Inhibiting FAT1 Blocks Metabolic Bypass to Enhance Antitumor Efficacy of TCA Cycle Inhibition through Suppressing CPT1A‐Dependent Fatty Acid Oxidation

**DOI:** 10.1002/advs.202502146

**Published:** 2025-05-23

**Authors:** Fanghui Chen, Jianqiang Yang, David O. Popoola, Fan Yang, Yajie Liu, Dongsheng Wang, Zhaohui S. Qin, Zhengjia Chen, Nabil F. Saba, Zhuo G. Chen, Yamin Li, Yong Teng

**Affiliations:** ^1^ Department of Hematology and Medical Oncology Winship Cancer Institute Emory University Atlanta GA 30322 USA; ^2^ Department of Pharmacology State University of New York Upstate Medical University Syracuse NY 13210 USA; ^3^ Department of Biostatistics and Bioinformatics Rolling School of Public Health Emory University Atlanta GA 30322 USA; ^4^ Division of Epidemiology and Biostatistics School of Public Health University of Illinois at Chicago Chicago IL 60612 USA; ^5^ Biostatistics Shared Resource University of Illinois Cancer Center Chicago IL 60612 USA; ^6^ Wallace H. Coulter Department of Biomedical Engineering Georgia Institute of Technology and Emory University Atlanta GA 30322 USA

**Keywords:** CPI‐613 sensitivity, CPT1A, FAT1, fatty acid oxidation, head and neck cancer, metabolic bypass

## Abstract

FAT atypical cadherin 1 (*FAT1*) is one of the most frequently mutated genes in head and neck squamous cell carcinoma (HNSCC), exhibiting the highest mutation rate across different tumor types. Although FAT1's role has attracted considerable attention, its impact on cancer metabolism and treatment resistance remains poorly understood. In this study, it is demonstrated that knockout of mutant *FAT1* in HNSCC cells attenuates CPT1A‐driven fatty acid oxidation (FAO) through downregulation of the transcription factor ASCL2, leading to marked suppression of tumor growth. Notably, *FAT1*‐mutant HNSCC cells exhibit resistance to the TCA cycle inhibitor CPI‐613 through activation of CPT1A‐mediated FAO, whereas genetic ablation of mutant *FAT1* restores sensitivity to CPI‐613. To achieve in vivo depletion of *FAT1*, LNP‐sgFAT1 is developed, a novel lipid nanoparticle (LNP) system encapsulating Cas9 mRNA and *FAT1*‐targeting sgRNA. In murine models bearing *FAT1*‐mutant head and neck tumors, LNP‐sgFAT1 demonstrated enhanced antitumor activity when combined with CPI‐613. Collectively, these findings establish that mutant *FAT1* drives CPT1A‐dependent FAO, facilitating a metabolic bypass that confers resistance to TCA cycle inhibition in HNSCC. This mechanistic insight highlights promising opportunities for combinatorial therapeutic strategies co‐targeting genetic and metabolic vulnerabilities in cancer.

## Graphical abstract

This study demonstrates that mutant FAT1 promotes ASCL2‐driven, CPT1A‐dependent fatty acid oxidation, leading to resistance to CPI‐613‐mediated TCA cycle inhibition in head and neck cancer. In vivo gene depletion of mutant FAT1 with LNP‐sgFAT1 suppresses tumor growth and restores CPI‐613 sensitivity, revealing a targetable metabolic bypass with therapeutic potential in FAT1‐mutant cancer. Figure created using Biorender (https://biorender.com/).

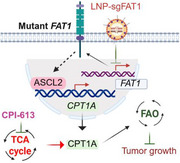

## 1 Introduction

The incidence of head and neck squamous cell carcinoma (HNSCC) in the United States has significantly increased in the past several decades with roughly 67,000 cases diagnosed yearly.^[^ ^1^ ^]^ Patients with HNSCC often experience a poor prognosis, and five‐year overall survival (OS) rates typically range from 30% to 70%, with variability influenced by factors such as tumor stage and location.^[^ ^2^ ^]^ Patients with recurrent/metastatic (R/M) HNSCC have a more limited OS despite recent advances in therapy.^[^ ^3^ ^]^ Most patients with R/M HNSCC have limited therapeutic options, and there is a lack of effective treatment approaches.^[^ ^4^, ^5^ ^]^ As the latest standard‐of‐care treatment strategy, immunotherapy with programmed cell death protein 1 (PD1) inhibitors (pembrolizumab and nivolumab) has been recently approved by the FDA to treat R/M HNSCC.^[^ ^1^ ^]^ However, the majority of HNSCC patients do not respond well to PD1‐based immunotherapy.^[^ ^6^, ^7^ ^]^ There is therefore an urgent need for the development of new therapeutics and strategies to augment the sensitivity of currently employed therapies for the treatment of this disease.

FAT atypical cadherin 1 (FAT1) is a cell surface adhesion protein known to play a role in the suppression of cell migration and polarity.^[^ ^8^, ^9^ ^]^ The *FAT1* gene is among the most frequently mutated genes in many types of cancer, particularly in HNSCC.^[^ ^10^, ^11^ ^]^ Mutant *FAT1* dysregulates multiple signaling pathways, including Hippo‐YAP/TAZ and Wnt/β‐catenin, resulting in enhanced cell proliferation and survival, ultimately driving tumor progression.^[^ ^10^, ^12^ ^]^ In murine models of skin squamous cell carcinoma and lung tumors, loss of *FAT1* was shown to enhance tumor initiation, progression, invasiveness, stemness and metastasis through the induction of a hybrid epithelial‐to‐mesenchymal transition (EMT) state.^[^ ^13^ ^]^ Our previous integrative bioinformatics and statistical analyses revealed a panel of genes and proteins associated with *FAT1* mutation in HNSCC and demonstrated the impact of *FAT1* alterations on HNSCC patient survival.^[^ ^14^ ^]^ Although defining and understanding of the role of mutant *FAT1* has garnered significant attention, surprisingly little is known about its regulatory network and its influence on HNSCC malignancy and treatment resistance.

In the present study, we report for the first time that loss of mutant *FAT1* in HNSCC cells leads to tumor remission. Specifically, genetic ablation of mutant *FAT1* suppresses the expression of carnitine palmitoyl transferase 1A (CPT1A) at the transcriptional level via achaete‐scute‐like 2 (ASCL2), resulting in decreased fatty acid oxidation (FAO). Moreover, we observed reduced sensitivity to the tricarboxylic acid (TCA) cycle inhibitor CPI‐613 in *FAT1*‐mutant HNSCC cells, a phenomenon mechanistically associated with increased FAO driven by CPT1A. Genetic depletion of mutant *FAT1* or treatment with the CPT1A‐specific inhibitor ST1326 significantly improved the antitumor efficacy of CPI‐613, suggesting that FAT1‐CPT1A signaling may facilitate a metabolic bypass that attenuates the effectiveness of CPI‐613 in HNSCC. As no direct inhibitors of FAT1 are available, we depleted the *FAT1* gene using lipid nanoparticles (LNPs) encapsulating Cas9 mRNA and sgRNA specifically targeting the *FAT1* gene. This approach showed enhanced antitumor activity when combined with CPI‐613 in murine models bearing mutant *FAT1* head and neck tumors, offering a promising avenue for more effective and tailored cancer treatments.

## 2 Results

### 2.1 Mutant FAT1 Functions as a Tumor Promoter in HNSCC

**Figure 1:**
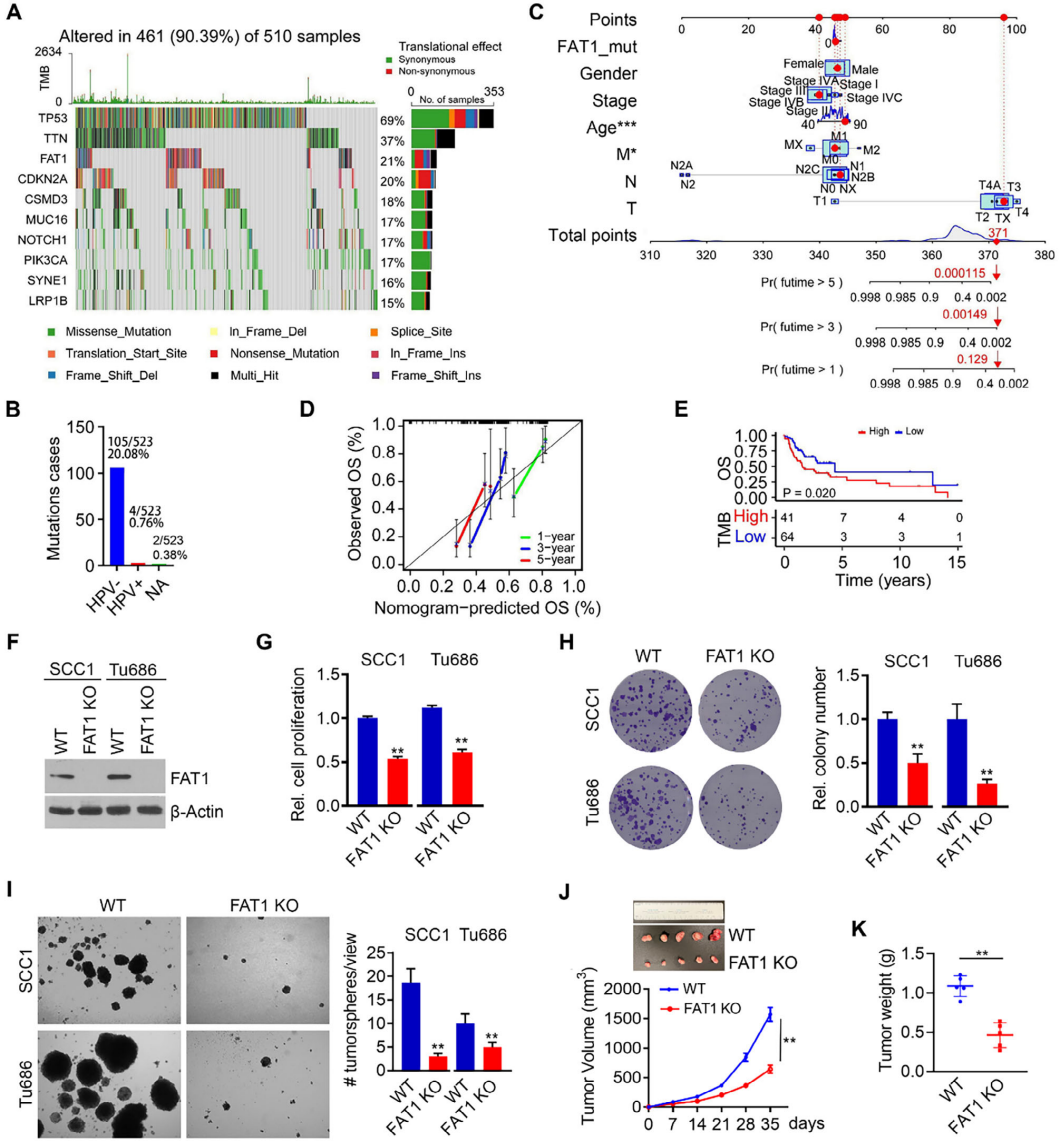
Mutant *FAT1* functions as a tumor promoter in HNSCC. A) Top 10 most frequently mutated genes in TCGA HNSCC cohort (n = 523). B) Frequency of *FAT1* mutation in HPV‐ and HPV+ tumor subtypes in TCGA HNSCC cohort. The number and percentage of cases with mutations are displayed. NA, not annotated. C) Clinical evaluation of *FAT1* mutations in HPV‐ tumors in TCGA HNSCC cohort. Points represent the individual score of each variable at different values, and total points represent the total score of the corresponding individual scores for all variables. Pr (prediction) represents the predicted overall survival (OS) at indicated years. D) Calibration curve assessing the reliability of nomogram model for OS in HPV‐ HNSCC patients with *FAT1* mutations. Calibration curve values greater than 0.75 suggest the credibility of the nomogram model. E) High TMB associated with *FAT1* mutations as a potential risk factor for OS in HPV‐ HNSCC patients with *FAT1* mutations in TCGA cohort. F) *FAT1* KO efficiency in SCC1 and Tu686 cells determined by Western blot. G–I) Effect of *FAT1* KO on cell proliferation (for 3 days), colony formation (for 14 days), and tumorsphere formation (for 14 days) in SCC1 and Tu686 cells. In (H) and (I), the experiments were performed in triplicate and quantitative data are shown in the right panel. (J, K) Representative flank tumors and tumor growth curve J), and tumor weight K) in the indicated treatment groups. *FAT1* KO or parental SCC1 cells were implanted to the right flank of 6‐week‐old NSG mice (n = 5/group), and tumor size was measured by a digital caliper weekly for 35 days. Bars express mean ± SD. Statistical analyses were conducted using unpaired two‐tailed Student's *t*‐test. *p < 0.05; **p < 0.01.

We conducted a comprehensive evaluation of the impact of genetic mutations in 523 HNSCC patients using a total of 87,968 mutation sites obtained from TCGA HNSCC dataset. This encompassed various mutation types, including 3’ Flank, 3’ UTR, 5’ Flank, 5’ UTR, Frame Shift Deletion (Del), Frame Shift Insertion (Ins), Missense Mutation, Multi Hit, Nonsense Mutation, In Frame Del, In Frame Ins, Splice Site, and Translation Start Site, across all mutant genes. This analysis unveiled the following top 10 mutant genes in HNSCC patients, with their respective mutation frequencies indicated in parentheses: *TP53* (69%), *TTN* (37%), *FAT1* (21%), *CDKN2A* (20%), *CSMD3* (18%), *MUC16* (17%), *NOTCH1* (17%), *PIK3CA* (17%), *SYNE1* (16%), and *LRP1B* (15%) (**Figure** **1**A). These gene mutations collectively accounted for 90.39% of the mutation sites observed in 461 patients out of 510 patients with all mutant genes examined in this study (Figure 1A).

*FAT1* emerged as one of the top three mutant genes in HNSCC, with significantly higher mutation frequency in HPV‐negative (‐) tumor subtype compared with HPV‐positive (+) tumor subtype (20.08% vs. 0.76%) in TCGA HNSCC cohort (Figure 1B). The analysis of clinical characteristics of HNSCC patients and *FAT1* mutations unveiled significant associations between *FAT1* mutations and patients’ age, ranging from 40 to 90 years, as well as the M stage (Figure 1C). This analysis also revealed that *FAT1* mutation was a promising predictor of poor outcome in HPV‐ HNSCC patients, as *FAT1* mutation was statistically significant in predicting 3‐year OS (P = 0.00149) and 5‐year OS (P = 0.000115) for this patient population (Figure 1C). The calibration curve was further used to assess the reliability of the nomogram model for OS in HPV‐ HNSCC patients with *FAT1* mutations. The values on the calibration curve for 3‐year and 5‐year OS were 0.79 and 0.77, respectively (Figure 1D), which were considered reliable as calibration curve values are greater than 0.75. In addition, high tumor mutation burden (TMB) associated with *FAT1* mutations correlated with poor OS in HPV‐ HNSCC patients in TCGA cohort (Figure 1E).

Given the notable clinical significance of *FAT1* mutations in HPV‐ HNSCC patients, we focused on exploring the impact of mutant *FAT1* in HPV‐ HNSCC cells in the following assays. Our previous sequencing data have shown that UM‐SCC1 (SCC1) and MDA686TU (Tu686) cells harbor mutations in the *FAT1* gene.^[^ ^11^ ^]^ To understand the necessary dependency of mutant *FAT1* in HNSCC cells, we performed a CRISPR/Cas9‐mediated gene KO of *FAT1* in SCC1 and Tu686 to create *FAT1* KO cells (Figure 1F). *FAT1* KO in these two cell lines significantly reduced cell proliferation and colony formation potential compared with the respective parental cells (Figure 1G,H). Furthermore, depletion of mutant *FAT1* resulted in a remarkable suppression of tumorsphere formation, as evidenced by a reduction in either tumorsphere size or number in *FAT1* KO cells compared to parental control cells (Figure 1I). In a flank‐xenograft mouse model, a significant reduction in tumor volume and weight (Figure 1J,K) was observed in mice implanted with *FAT1* KO cells compared to mice receiving parental cells. In contrast, knockout of *FAT1* in HN12 and JHU029 cells, which harbor wild‐type *FAT1*, resulted in enhanced cell proliferation and increased colony formation (Figure S1, Supporting Information), highlighting the tumor‐suppressive role of wild‐type *FAT1* in HNSCC cells.

### 2.2 Loss of Mutant FAT1 Downregulates FAO‐Associated Signaling in HNSCC Cells

**Figure 2:**
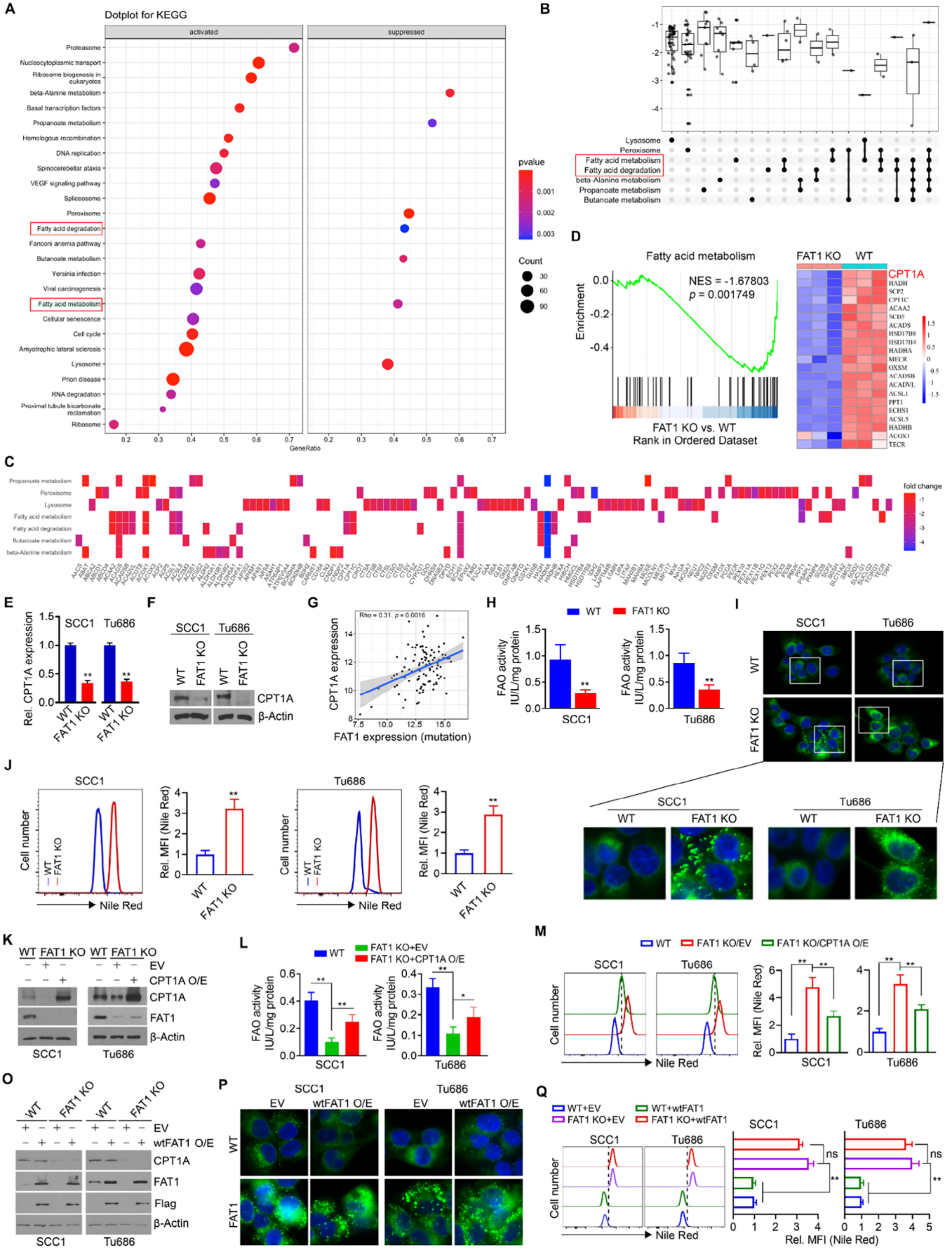
Depletion of mutant *FAT1* downregulates FAM‐associated signaling in HNSCC cells. A–D) Gene expression profile and pathways analysis based on RNA‐seq data from *FAT1* KO and parental SCC1cells. A) A dot plot of enriched KEGG pathways based on DEGs identified in *FAT1* KO SCC1 cells vs. parental cells. The signaling pathways associated with FAM are outlined in red. B) An upset plot of enriched GO terms based on downregulated genes in *FAT1* KO SCC1cells vs. parental cells. Each point represents the log2 *FC* of each gene in a gene set. The greatest valued points are the most downregulated genes enriched in the given biological processes. The signaling pathways associated with FAM are outlined in red. C) Heatmap analysis of DEGs identified in *FAT1* KO SCC1 cells vs. parental cells. D) DEGs in FAM signaling were tested for enrichment via Gene Set Enrichment Analysis (GSEA). The panel to the right of GSEA plot shows the corresponding heatmap of DEGs in FAM signaling. E,F) Decreased CPT1A expression confirmed by qRT‐PCR and Western blot in *FAT1* KO and parental SCC1 and Tu686 cells. G) A positive correlation between the expression of mutant *FAT1* and CPT1A illustrated in scatter plot using the RNA‐seq data from TCGA HNSCC cohort. H) Relative quantification of FAO in SCC1 and Tu686 cells with or without *FAT1* KO determined by FAO activity assay. I) Representative fluorescence confocal microscopy images of BODIPY 493/503 staining (green) in *FAT1* KO and parental SCC1 and Tu686 cells. J) Representative images and quantitative data of flow cytometry analysis using Nile Red staining in *FAT1* KO and parental SCC1 and Tu686 cells. K) *CPT1A* overexpression (CPT1A O/E) in *FAT1* KO SCC1 and Tu686 cells. L,M) Effect of restoring *CPT1A* expression on FAO levels in *FAT1* KO SCC1 and Tu686 cells. The results from FAO activity assay and flow cytometry analysis using Nile Red staining are shown in (L) and (M), respectively. O) Effect of wtFAT1 overexpression on CPT1A levels in *FAT1* KO and parental SCC1 and Tu686 cells. P,Q) Effect of wtFAT1 overexpression on LD formation in *FAT1* KO and parental SCC1 and Tu686 cells. Representative images of BODIPY 493/503 staining and quantitative data of flow cytometry analysis using Nile Red staining are shown in (P) and (Q), respectively. Bars express mean ± SD. Statistical analyses were conducted using unpaired two‐tailed Student's *t*‐test. **p < 0.01.

To explore changes in gene expression occurring in HNSCC cells with or without loss of mutant *FAT1*, we performed RNA‐seq analysis using RNA samples derived from *FAT1* KO and parental SCC1 cells. The dot plot illustrating enriched KEGG pathways based on differentially expressed genes (DEGs) identified in our RNA‐seq data revealed a downregulation of pathways associated with fatty acid metabolism (FAM) in SCC1 cells when *FAT1* was knocked out (**Figure** **2**A). This observation was consistent with the results obtained from the analysis of enriched GO terms (Figure 2B). The ridge plot further showed gene expression distribution in the FAM‐related gene set (Figure S2, Supporting Information). We also performed heatmap and gene network analysis of DEGs and found that several FAM‐related genes, including *CPT1A*, exhibited downregulation in *FAT1* KO SCC1 cells compared with parental cells (Figure 2C; Figure S3, Supporting Information). DEGs associated with FAM signaling were further enriched via gene set enrichment analysis (GSEA), and this analysis revealed *CPT1A*, a gene encoding the key rate‐limiting enzyme of FAM, as the top downregulated gene involved in FAM signaling in *FAT1* KO SCC1 cells (Figure 2D). Dramatically decreased CPT1A expression was confirmed by qRT‐PCR and Western blot upon *FAT1* KO in both SCC1 and Tu686 cells (Figure 2E,F). However, depletion of *FAT1* in HN12 and JHU029 cells harboring wild‐type *FAT1* did not lead to a noticeable reduction in CPT1A levels (Figure S4, Supporting Information). Moreover, our bioinformatics analysis revealed a robust correlation between the gene expression of mutant *FAT1* and *CPT1A* in TCGA HNSCC cohort (Figure 2G, Rho = 0.31, *p* = 0.0016).

CPT1A plays a critical role in the regulation of FAO, which is a specific process within the broader field of FAM.^[^ ^15^ ^]^ Since mutant *FAT1* regulates CTP1A expression in HNSCC cells, we sought to understand whether depleting mutant *FAT1* in HNSCC cells affects FAO levels. Intriguingly, FAO activity assay showed a significant reduction in SCC1 and Tu686 cells upon *FAT1* KO (Figure 2H). To corroborate this result, we performed lipid droplet (LD) staining of *FAT1* KO and parental SCC1 and Tu686 cells. BODIPY 493/503 and Nile Red are excellent vital stains for the detection and quantification of intracellular LDs. *FAT1* KO cells displayed a significantly stronger staining intensity for both BODIPY 493/503 and Nile Red compared to their parental controls (Figure 2I,J), suggesting a reduction in fatty acid utilization for energy production upon *FAT1* KO. To determine the contribution of CPT1A in FAT1‐mediated FAO, we overexpressed *CPT1A* in *FAT1* KO cells (Figure 2K). Reintroducing *CPT1A* expression reversed reduced FAO levels (Figure 2L) and attenuated accumulated LDs (Figure 2M) in SCC1 and Tu686 following *FAT1* KO. In addition, this restoration resulted in an elevation of cell proliferation and enhanced colony formation in *FAT1* KO cells compared with *FAT1* KO cells expressing empty vector (EV) (Figure S5, Supporting Information). To further determine whether wild‐type *FAT1* affected CPT1A expression and its mediated FAO, we overexpressed human full‐length wild‐type *FAT1* plasmid (wtFAT1 O/E) in *FAT1* KO SCC1 and Tu686 cells. This overexpression did not rescue mutant *FAT1* KO‐mediated decrease in CPT1A expression (Figure 2O) and accumulated LD formation (Figure 2P,Q), supporting the notion that mutant *FAT1*, but not wild‐type *FAT1*, promotes FAO by upregulating *CPT1A* in HNSCC cells.

### 2.3 Loss of Mutant FAT1 Downregulates CPT1A Expression Levels in HNSCC Cells through Suppressing ASCL2

**Figure 3:**
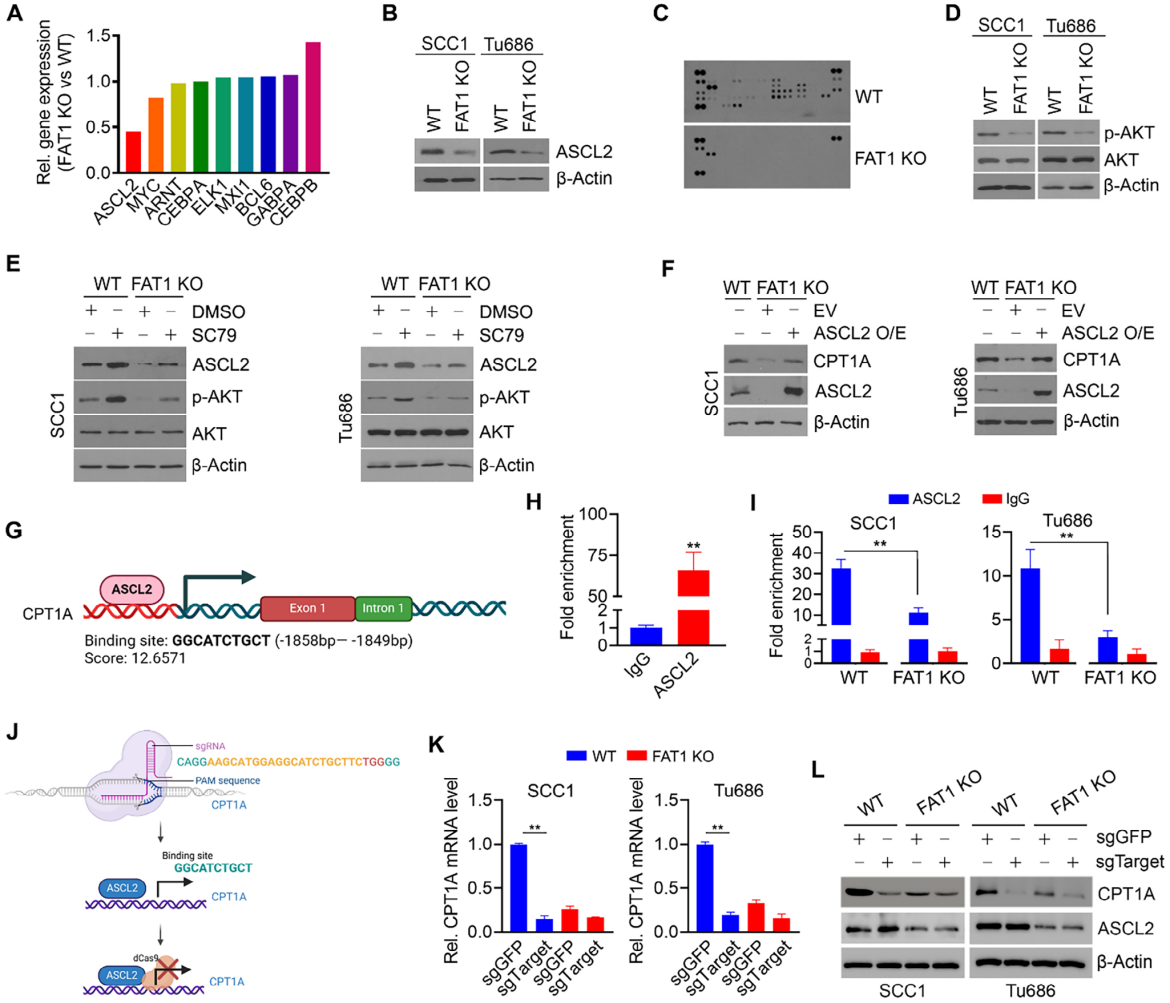
Loss of mutant *FAT1* downregulates *CPT1A* gene expression in HNSCC cells through suppressing ASCL2. A) Fold changes of the downregulated transcription factor genes in *FAT1* KO vs. parental SCC1 cells determined by RNA‐seq data. B) Alterations in ASCL2 protein levels between *FAT1* KO and parental SCC1 and Tu686 cells determined by Western blot. C) Effect of *FAT1* KO on the tyrosine phosphorylation levels of RTKs determined by Human Phospho‐RTK array in SCC1 cells. D) Effect of *FAT1* KO on the p‐AKT levels in SCC1 and Tu686 cells. E) Effect of AKT activator SC79 on *FAT1* KO‐mediated reduction of ASCL2 expression in SCC1 and Tu686 cells. F) Effect of restoring *ASCL2* expression on *FAT1* KO‐mediated reduction of CPT1A expression in SCC1 and Tu686 cells. G) Schematic of the DNA binding site of ASCL2 on the upstream promoter of the *CPT1A* gene. H) Binding of ASCL2 to the *CPT1A* gene promoter in SCC1 cells determined by ChIP assay. I) Changes in the binding amount of ASCL2 to the *CPT1A* gene promoter in *FAT1* KO and parental SCC1 and Tu686 cells determined by ChIP‐qPCR analysis. J) Schematic showing the sgTarget that specifically targets ASCL2 binding site at the *CPT1A* gene promoter. K,L) Effect of the sgTarget on CPT1A expression (K) and protein (L) levels in in *FAT1* KO and parental SCC1 and Tu686 cells. *FAT1* KO and parental SCC1 and Tu686 cells were co‐transfected with dCas9 and sgTarget or sgGFP, and the CPT1A expression and protein levels were determined by qRT‐PCR and Western blot. Bars express mean ± SD. Statistical analyses were conducted using unpaired two‐tailed Student's *t*‐test. *p < 0.05; **p < 0.01.

To investigate the molecular mechanism by which FAT1 regulates CPT1A in HNSCC cells, we identified 9 DEGs that functioned as transcription factors based on the RNA‐seq data from *FAT1* KO and parental SCC1 cells. Among them, *ASCL2* was the most downregulated transcription factor in *FAT1* KO cells compared to parental cells (**Figure** **3**A). Western blot analysis confirmed reduced ASCL2 levels in *FAT1* KO SCC1 and Tu686 cells (Figure 3B). However, no change in ASCL2 levels was observed in HN12 and JHU029 cells, regardless of *FAT1* KO (Figure S4, Supporting Information). Notably, when we examined alterations in receptor tyrosine kinase (RTK) phosphorylation in SCC1 cells with or without *FAT1* KO, we found a widespread reduction in phosphorylation across all 42 RTKs assessed using the Proteome Profiler Human Phospho‐RTK Array following *FAT1* KO (Figure 3C). Similarly, phosphorylation of AKT, a critical downstream effector of RTKs, was downregulated in *FAT1* KO cells compared to parental cells (Figure 3D). To investigate whether AKT mediates the regulation of ASCL2 by FAT1, we reintroduced AKT activation in *FAT1* KO SCC1 and Tu686 cells using the AKT activator SC79. This restoration of AKT activity successfully recovered ASCL2 levels in these cells (Figure 3E), demonstrating that FAT1 modulates ASCL2 via the AKT signaling pathway. Most importantly, restoration of *ASCL2* expression in *FAT1* KO SCC1 and Tu686 cells rescued CPT1A expression (Figure 3F), suggesting the requirement for ASCL2 in FAT1‐mediated CPT1A regulation.

Promoter analysis identified a single consensus ASCL2 binding site spanning ‐1849 bp and ‐1858 bp on the *CPT1A* gene promoter (Figure 3G). Chromatin immunoprecipitation followed by qPCR analysis (ChIP‐qPCR) revealed a distinct ASCL2 occupancy on the *CPT1A* gene promoter (Figure 3H) and a significant decrease in the binding amount of ASCL2 to the *CPT1A* gene promoter in both SCC1 and Tu686 cells with *FAT1* KO (Figure 3I). To determine the importance of the ASCL2 binding site within transcriptional networks, CRISPRd was used to disrupt ASCL2 binding to the *CPT1A* gene promoter.^[^ ^16^ ^]^ A sgRNA specifically targeting the ASCL2 binding site on the *CPT1A* gene promoter (sgTarget) was co‐transfected with deactivated Cas9 (dCas9) vector p‐LV‐TRE3G‐dCas9‐DsRed‐Zeo (Figure 3J). Disruption of the ASCL2 binding site on the *CPT1A* gene promoter dramatically suppressed *CPT1A* expression and protein levels in both SCC1 and Tu686 cells (Figure 3K,L). Further decreased CPT1A expression was observed in *FAT1* KO cells when the ASCL2 binding site on the *CPT1A* gene promoter was disrupted (Figure 3H,I). Moreover, restoring *ASCL2* or *CPT1A* expression in *FAT1* KO SCC1 cells not only counteracted the reduction in cell proliferation and clonogenicity mediated by *FAT1* KO (Figure S6, Supporting Information), but also reversed the inhibition of tumor growth in an orthotopic mouse model (Figure S7, Supporting Information). These findings demonstrate the dependency of *CPT1A* expression on ASCL2 activity and highlight that ASCL2 is required for the regulation of CPT1A by mutant *FAT1* in HNSCC cells.

### 2.4 Mutant FAT1 Decreases Drug Sensitivity of HPV‐ HNSCC to TCA Cycle Inhibitors

**Figure 4:**
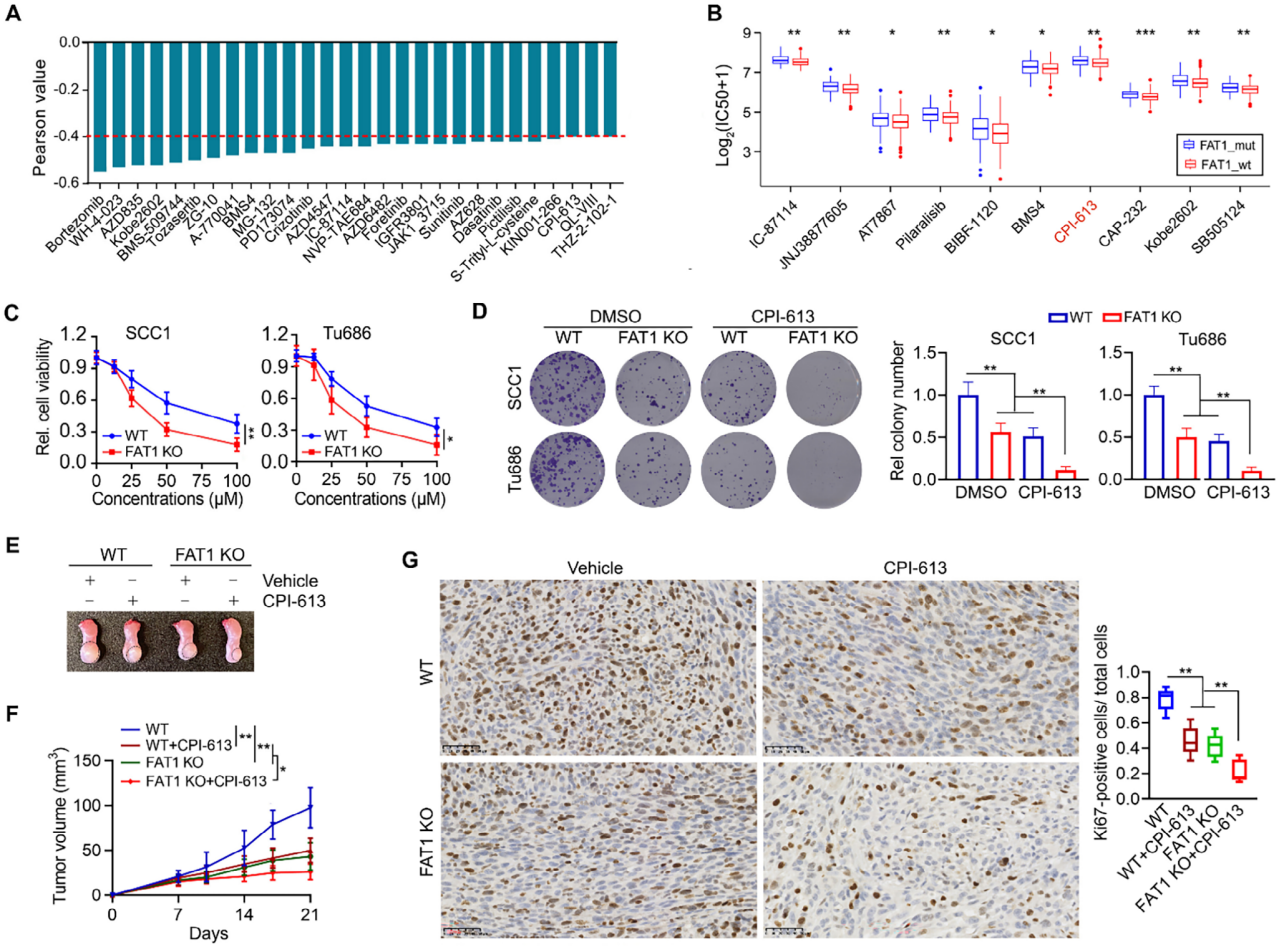
*FAT1* mutations reduce the sensitivity of HPV‐negative HNSCC to CPI‐613. A) Pearson correlation analysis between the expression of the mutant *FAT1* gene in HPV‐ tumor cases and the IC50 values of drugs available in GDSCs led to the identification of 28 drugs meeting our screening criteria (Spearman correlation value≤‐0.4, P< 0.05). B) Top 10 drugs showing significantly differential sensitivity in HPV‐ HNSCC cases carrying mutant *FAT1* compared to those carrying wild‐type *FAT1*. C,D) Effect of *FAT1* KO on sensitivity to CPI‐613 in SCC1 and Tu686 cells determined by cell viability (C) and colony formation (D). E,F) Effect of *FAT1* KO on the antitumor activity of CPI‐613 in orthotopic tongue tumor mice. Representative tongue tumors (E) and tumor growth curve (F) in the indicated treatment groups. *FAT1* KO or parental SCC1 cells were implanted to the tongue of 6‐week‐old NSG mice (n = 5/group), and 50 mg kg^−1^ CPI‐613 was administered by *i.p*. injection on day 10 and was given once daily for 10 days. Tumor size was measured by a digital caliper twice per week for 21 days. G) IHC with anti‐Ki67 antibody in *FAT1* KO and parental SCC1 tumors with or without CPI‐613 treatment. Representative IHC imaging and quantitative data are shown in the left and right panels. Bars express mean ± SD. Statistical analyses were conducted using unpaired two‐tailed Student's *t*‐test. *p < 0.05; **p < 0.01; and ***p < 0.001.

To investigate the impact of *FAT1* mutations on drug responsiveness in HPV‐ HNSCC, we obtained expression data from TCGA for HPV‐ HNSCC with *FAT1* mutations. Subsequently, we examined the correlation between the expression levels of mutant *FAT1* in these cases and the IC50 values of drugs accessible in Genomics of Drug Sensitivity in Cancer (GDSC). This analysis revealed that the sensitivity to 28 drugs (e.g., bortezomib, WH‐4‐023, AZD8825 and CPI‐613) exhibited a significantly negative association (Spearman correlation value ≤ ‐0.4, *p*<0.05) with the elevated expression of the mutant *FAT1* gene in HPV‐ HNSCC (**Figure** **4**A). Furthermore, we conducted an analysis of the IC50 values of GDSC drugs within two subgroups of HPV‐ HNSCC, stratified based on *FAT1* mutation status (wild‐type vs. mutations). Remarkably, CPI‐613 was identified as one of the top 10 drugs showing significant differences in sensitivity between HPV‐ HNSCC cases with mutant *FAT1* and those with wild‐type *FAT1* (Figure 4B). This bioinformatic discovery was supported by cell viability assays showing that HNSCC cell lines with mutant *FAT1* were less sensitive to CPI‐613 compared to those with wild‐type *FAT1* (Figure S8, Supporting Information).

To verify the impact of mutant *FAT1* on sensitivity to CPI‐613, we treated *FAT1* KO and parental SCC1 and Tu686 cells with CPI‐613. CPI‐613 is an innovative systemic therapeutic agent inhibiting the TCA cycle,^[^ ^17^, ^18^ ^]^ and we found that CPI‐613 inhibited cell viability and clonogenicity more efficiently in *FAT1* KO cells compared to parental cells (Figure 4C,D). There was no difference in cell viability between *FAT1* KO and parental HN12 and JHU029 cells when treated with the same dosages of CPI‐613 (Figure S9, Supporting Information). Consistently, malonate and lonidamine, two other TCA cycle inhibitors, also demonstrated enhanced anticancer effects in SCC1 and Tu686 cells following *FAT1* KO (Figure S10, Supporting Information). These findings suggest that mutant *FAT1* may reduce the sensitivity of HNSCC to TCA cycle inhibitors.

To further evaluate whether loss of mutant *FAT1* affects the efficacy of CPI‐613 treatment in vivo, *FAT1* KO and parental SCC1 cells were individually injected into the anterior tongue of NSG mice, generating an orthotopic tongue tumor mouse model. Notably, there was a significant reduction in tumor volume and a marked decrease in Ki67‐positive tumor cells in mice implanted with *FAT1* KO cells compared to those with parental cells (Figure 4E–G). Importantly, the additional loss of *FAT1* enhanced the antitumor activity of CPI‐613 treatment in SCC1 tumors, as evidenced by a further decrease in tongue tumor growth (Figure 4E,F) and a reduced number of Ki67‐positive cells in tumor tissues (Figure 4G). These findings suggest that inhibiting mutant *FAT1* could improve the sensitivity of HNSCC cells to CPI‐613 treatment.

### 2.5 Depletion of Mutant *FAT1* Enhances the Sensitivity of HNSCC Cells to CPI‐613 by Engaging a Mechanism that Depends on CPT1A‐Mediated FAO

**Figure 5:**
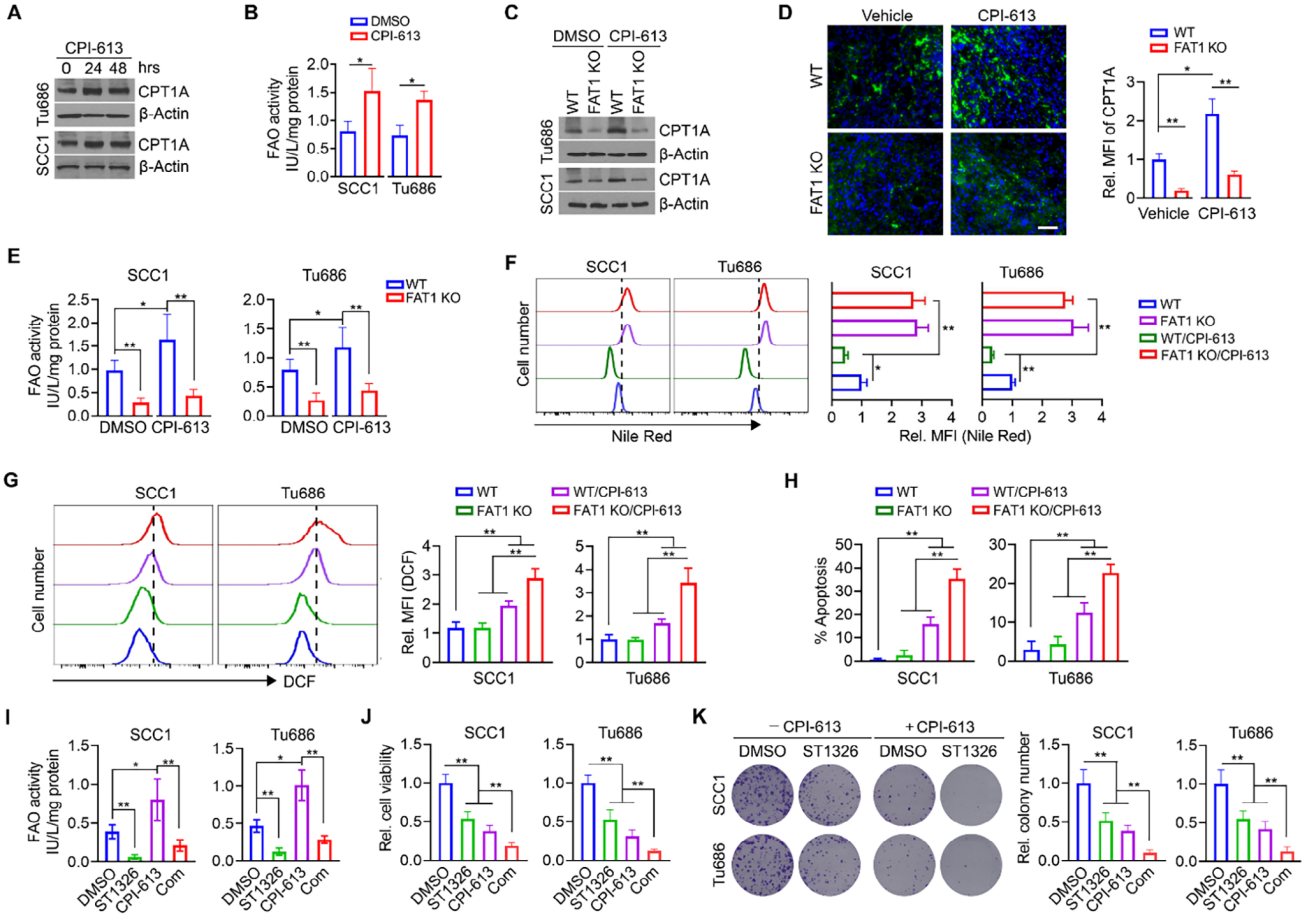
Mutant *FAT1* contributes to reduced sensitivity of HNSCC cells to CPI‐613 via CPT1A‐dependent FAO. A) CPT1A levels in SCC1 and Tu686 cells treated with 50 µm CPI‐613 for 24 and 48 h. B) FAO levels in SCC1 and Tu686 cells with or without 50 µm CPI‐613 for 24 h determined by FAO activity assay. C) Effect of *FAT1* KO on CPT1A expression in SCC1 and Tu686 cells treated with or without 50µM CPI‐613 for 24 h. D) IF with anti‐CPT1A antibody in *FAT1* KO and parental SCC1 tumors with or without CPI‐613 treatment. Representative IF imaging and quantitative data are shown in the left and right panels. Scale bar = 50 µm. E) Effect of CPI‐613 on FAO levels in *FAT1* KO and parental SCC1 and Tu686 cells. F) Representative images and quantitative data of flow cytometry analysis (n = 3) using Nile Red staining in *FAT1* KO and parental SCC1 and Tu686 cells, in the presence or absence of 50 µm CPI‐613 for 24 h. G) Representative images and quantitative data of flow cytometry analysis (n = 3) using DCF staining in *FAT1* KO and parental SCC1 and Tu686 cells, in the presence or absence of 50µM CPI‐613 for 24 h. H) Apoptosis determined by flow cytometry analysis (n = 3) after Annexin V/7‐AAD double staining in *FAT1* KO and parental SCC1 and Tu686 cells, in the presence or absence of 50 µm CPI‐613 for 48 h. I–K) Effect of the CPI‐613/ST1326 combination on FAO activity (I), cell viability (J) and colony formation (K) of SCC1 and Tu686 cells. Cells were treated with DMSO, 50 µm CPI‐613 and 5 µm ST1326, alone or in combination. Bars express mean ± SD. Statistical analyses were conducted using two‐tailed unpaired Student's *t*‐test. *p < 0.05; **p < 0.01; and ***p < 0.001.

Our bioinformatics analysis revealed a robust correlation between the expression of *CPT1A* and the sensitivity to CPI‐613 treatment in HNSCC cases harboring *FAT1* mutations, whereas no such correlation was observed in HNSCC cases with wild‐type *FAT1* (Figure S11, Supporting Information). Next, we sought to study whether the FAT1‐CPT1A signaling axis played a role in altering CPI‐613 sensitivity in HNSCC cells harboring mutant *FAT1*. To explore this, we assessed CPT1A levels in SCC1 and Tu686 cells following CPI‐613 exposure. Our analysis revealed that CPT1A levels were elevated in both cell lines following CPI‐613 treatment (**Figure** **5**A), which coincided with an increase in FAO activity (Figure 5B). Notably, increased CPT1A levels were also observed in SCC1 and Tu686 cells treated with either malonate or lonidamine (Figure S12, Supporting Information), suggesting that TCA cycle inhibition may induce enhanced CPT1A expression in HNSCC cells harboring mutant *FAT1*. Furthermore, depletion of *FAT1* in SCC1 and Tu686 cells led to a reduction in CPT1A levels in the presence of CPI‐613 (Figure 5C). These findings were validated through immunofluorescence (IF) analysis using an anti‐CPT1A antibody in *FAT1* KO and parental SCC1 tumors (Figure 5D). Additionally, *FAT1* KO resulted in the decrease of the elevated FAO in SCC1 and Tu686 cells treated with CPI‐613 (Figure 5E,F). Fatty acid utilization for mitochondrial respiration was measured by analyzing oxygen consumption rate (OCR). A marked decrease in OCR was observed following etomoxir (Eto) injection in CPI‐613‐treated SCC1 cells, indicating that these cells primarily depend on FAO for respiration (Figure S13, Supporting Information). However, no change in OCR was noted in *FAT1* KO cells after Eto injection, regardless of CPI‐613 treatment, suggesting that mitochondrial respiration in these cells occurs independently of FAO (Figure S13, Supporting Information). FAO inhibition can result in reduced NADPH and GSH levels, increasing cellular susceptibility to oxidative stress.^[^ ^19^ ^]^ In line with this, 2’,7’‐dichlorofluorescein (DCF) staining analysis revealed that *FAT1* KO combined with CPI‐613 led to a greater increase in ROS levels in SCC1 and Tu686 cells compared to CPI‐613 treatment alone (Figure 5G), leading to higher rates of apoptosis (Figure 5H). These findings suggest that the depletion of mutant *FAT1* sensitizes HNSCC cells to CPI‐613, at least in part, through ROS‐associated apoptosis via FAO suppression.

ST1326, a selective CPT1A inhibitor, has an oral formulation (Teglicar) and has been tested in Phase 2 studies for the treatment of type 2 diabetes, where it demonstrated an excellent safety profile.^[^ ^20^, ^21^ ^]^ We then treated SCC1 and Tu686 cells with CPI‐613 and ST1326, alone or in combination. ST1326 treatment alone dramatically suppressed FAO activity, leading to decreased cell viability and colony formation (Figure 5I,K). Most notably, ST1326 effectively reduced the CPI‐613‐induced increase in FAO activity (Figure 5I). Consequently, combining ST1326 with CPI‐613 enhanced the inhibitory effects on cell viability and colony formation compared to either treatment alone in both cell lines (Figure 5J,K), suggesting that targeting FAO in conjunction with CPI‐613 could be a promising strategy to amplify its anti‐cancer effects.

### 2.6 Delivery of CRISPR/Cas9 mRNA with LNPs Facilitates FAT1 Gene Depletion in HNSCC Cells

**Figure 6:**
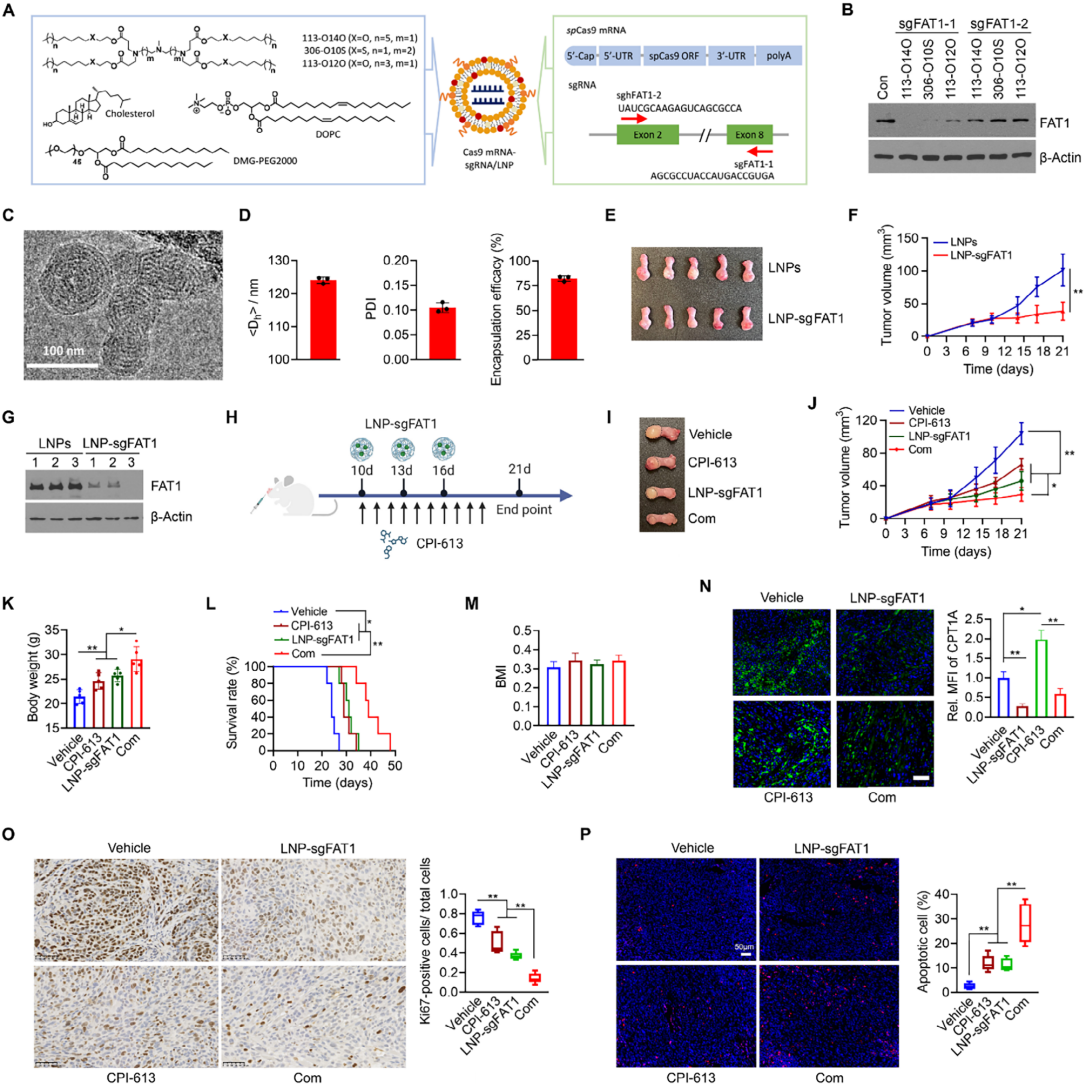
The combination of LNP‐sgFAT1 and CPI‐613 exhibits greater antitumor activity than either agent alone in orthotopic mice bearing head and neck tumors with *FAT1* mutation. A) Schematic representation of the (left) chemical structures of active lipids (113‐O14O, 306‐O10S, and 113‐O12O), cholesterol, DOPC, and DMG‐PEG2k, and (right) structure of spCas9 mRNA and sequences of sgRNA targeting exon 2 (sgFAT1‐1) and exon 8 (sgFAT1‐2) in the human *FAT1* gene. B) KO efficiency of sgFAT1 co‐delivered with Cas9 mRNA by different LNP(s) (113‐O14O, 306‐O10S, and 113‐O12O) in SCC1 cells. Western blot analysis was performed after 72 h of exposure to different LNP formulations. C) Representative cryoEM image of Cas9 mRNA‐sgRNA‐LNP. D) Hydrodynamic size, polydispersity index (PDI) and encapsulation efficacy of LNP‐sgFAT1. E) Tongues with SCC1 tumors extracted from NSG mice treated with control LNP or LNP‐sgFAT1. F) Tumor growth curve in SCC1 tongue tumor‐bearing mice treated with control LNP or LNP‐sgFAT1. G) KO efficiency of LNP‐sgFAT1 in SCC1 tumors determined by Western blot. H) Experimental procedure for in vivo studies. SCC1 tumor‐bearing NSG mice were treated with CPI‐613 and LNP‐sgFAT1, alone or in combination. In this study, 50 mg kg^−1^ CPI‐613 was administered by *i.p*. injection every day for 10 days, and 5 mg kg^−1^ LNP‐sgFAT1 was administered by intratumoral injection every three days for three doses. Tumor size was measured two or three times per week for 21 days (n = 5 mice per group). I–K) Representative tumors (I), tumor growth curve (J), and body weight (K) in each treatment group. L) Kaplan‐Meier plot with log‐rank test for survival for mice treated with or without CPI‐613 and/or LNP‐sgFAT1 (n = 5 mice per group). M) The body mass index (BMI) of mice treated with or without CPI‐613 and/or LNP‐sgFAT1 (n = 5 mice pergroup). N) IF with anti‐CPT1A antibody in SCC1 tumors following single or combination treatment, as indicated. Representative IF imaging and quantitative data are shown in the left and right panels. Scale bar = 50 µm. O,P) IHC of Ki67 and TUNEL assays using the tumor tissues collected from each treatment group. Representative results and quantitative data are shown in the left and right panels. Bars express mean ± SD. Statistical analyses were conducted using unpaired two‐tailed Student's *t*‐test. *p < 0.05; **p < 0.01.

Currently, no small molecule drugs or inhibitors specifically target FAT1. We thus engineered LNPs for CRISPR/Cas9‐mediated *FAT1* depletion in vitro and in vivo. Following our previously reported procedures,^[^ ^22^ ^]^ chalcogen‐containing ionizable lipids (113‐O14O, 306‐O10S, and 113‐O12O) were synthesized by reacting polyamine head (113 and 306) with chalcogen ether hydrophobic tails (O14O, O10S, and O12O) through the Michael addition reaction. The products were purified by a Teledyne Isco Chromatography purification system using DCM and MeOH as the mobile phase. Blank LNPs were prepared by self‐assembling ionizable lipids with cholesterol, DOPC, and DMG‐PEG2k in sodium acetate buffer using the solvent exchange method (**Figure** **6**A).^[^ ^23^, ^24^ ^]^ In this formulation, cholesterol stabilizes the nanoparticle, regulates membrane rigidity and fluidity, and aids bilayer fusion. DOPC supports LNP formation and endosomal escape, while DMG‐PEG2k prevents aggregation and non‐specific absorption.^[^ ^25^, ^26^ ^]^ Following dialysis, blank LNPs were combined with SpCas9 mRNA and sgRNA at a 16/1/1 weight ratio (LNP/mRNA/sgRNA) to generate cargo‐loaded LNPs, termed Cas9 mRNA‐sgFAT1/LNP (Figure 6A). Two FAT1‐targeting sgRNAs (sgFAT1‐1 targeting exon 8 and sgFAT1‐2 targeting exon 2) were loaded into LNPs via physical complexation, resulting in six distinct mRNA‐sgRNA/LNP formulations (Figure 6A). These LNP formulations were individually applied to treat SCC1 cells. Among them, Cas9 mRNA‐sgFAT1‐1/113‐O14O resulted in the most efficient FAT1 depletion at protein levels after 72 h of exposure (Figure 6B). The newly developed Cas9 mRNA‐sgFAT1‐1/113‐O14O was referred to as LNP‐sgFAT1. Sanger sequencing and Indel analysis validated the successful *FAT1* KO in SCC1 cells treated with LNP‐sgFAT1 (Figure S14, Supporting Information). In contrast to LNP‐sgFAT1, blank LNPs and Cas9‐LNPs loaded with a scrambled sgRNA (LNP‐sgNC) had no impact on *FAT1* gene depletion, cell proliferation, or colony formation (Figure S15, Supporting Information), suggesting that the effect observed with LNP‐sgFAT1 is specific to *FAT1* targeting. Cryogenic electron microscopy (cryoEM) revealed a spherical, onion‐like multi‐lamellar structure for LNP‐sgFAT1 (Figure 6C), a common morphology for RNA‐loaded LNPs.^[^ ^27^, ^28^ ^]^ Similar to the well‐characterized siRNA‐LNP complex, it is speculated that the mRNA and sgRNA molecules in our system were likely sandwiched between the positively charged bilayer lipid membranes.^[^ ^29^, ^30^ ^]^ DLS analysis revealed that the average hydrodynamic diameter (<*D*_h_>) of LNP‐sgFAT1 was 124.0 (Figure 6D), a size range considered optimal for intracellular delivery.^[^ ^31^, ^32^ ^]^ Moreover, LNP‐sgFAT1 had an average polydispersity index (PDI) of 0.11 (Figure 6D), indicating uniform particle size with no large aggregates. RiboGreen assay showed 82.3% RNA encapsulation (Figure 6D), suggesting an efficient RNA complexation driven by electrostatic interactions between positively charged LNPs and negatively charged RNA.^[^ ^28^, ^33^ ^]^

To determine the treatment efficacy of LNP‐sgFAT1, an orthotopic tongue tumor model was generated by injection of SCC1 cells into tongue of NSG mice. When tongue tumors were established, LNP‐sgFAT1 or its control blank LNP was administered by intratumoral injection every three days for three doses. On day 21, a significant reduction in tumor burden was observed following treatment with LNP‐sgFAT1 (Figure 6E,F). A significant reduction in FAT1 levels was observed in tumors treated with LNP‐sgFAT1 compared to those treated with control blank LNPs (Figure 6G), indicating an on‐target effect.

### 2.7 Co‐Administration of LNP‐sgFAT1 and CPI‐613 Demonstrates Enhanced Antitumor Efficacy Compared to Either Agent Alone in Orthotopic Mice Bearing Head and Neck Tumors with Mutant *FAT1*

To investigate whether further *FAT1* depletion could boost CPI‐613's antitumor activity, we treated SCC1 tongue tumor‐bearing NSG mice with CPI‐613 and LNP‐sgFAT1, either alone or in combination, following the treatment schedule outlined in Figure 6H. Treatment with either CPI‐613 or LNP‐sgFAT1 resulted in a significant reduction in tumor burden (Figure 6I,J). Consistent with in vitro findings, the combination of CPI‐613 and LNP‐sgFAT1 showed more effective tumor growth suppression in mice than either treatment alone (Figure 6I,J). Mice treated with vehicle showed a significant body weight loss compared to those treated with single agents or the combination (Figure 6K), likely due to larger tongue tumors affecting their eating and drinking. Survival curves demonstrated that the combination treatment prolonged survival in tumor‐bearing mice compared to the other three treatment groups (Figure 6L).

Given that CPI‐613 can potentially enhance FAO, we evaluated the body mass index (BMI) of mice receiving different treatments. The analysis revealed no significant differences in BMI across the groups (Figure 6M). However, in tumor tissues, an increase in CPT1A was observed after CPI‐613 treatment, which was reduced with the addition of LNP‐sgFAT1 (Figure 6N). At the molecular level, SCC1 tumors treated with both agents showed fewer Ki67‐positive cells compared to the single‐agent and control LNP groups (Figure 6O). Additionally, combination treatment led to a significant increase in apoptosis, as indicated by TUNEL assays, compared to the other treatments (Figure 6P).

**Figure 7:**
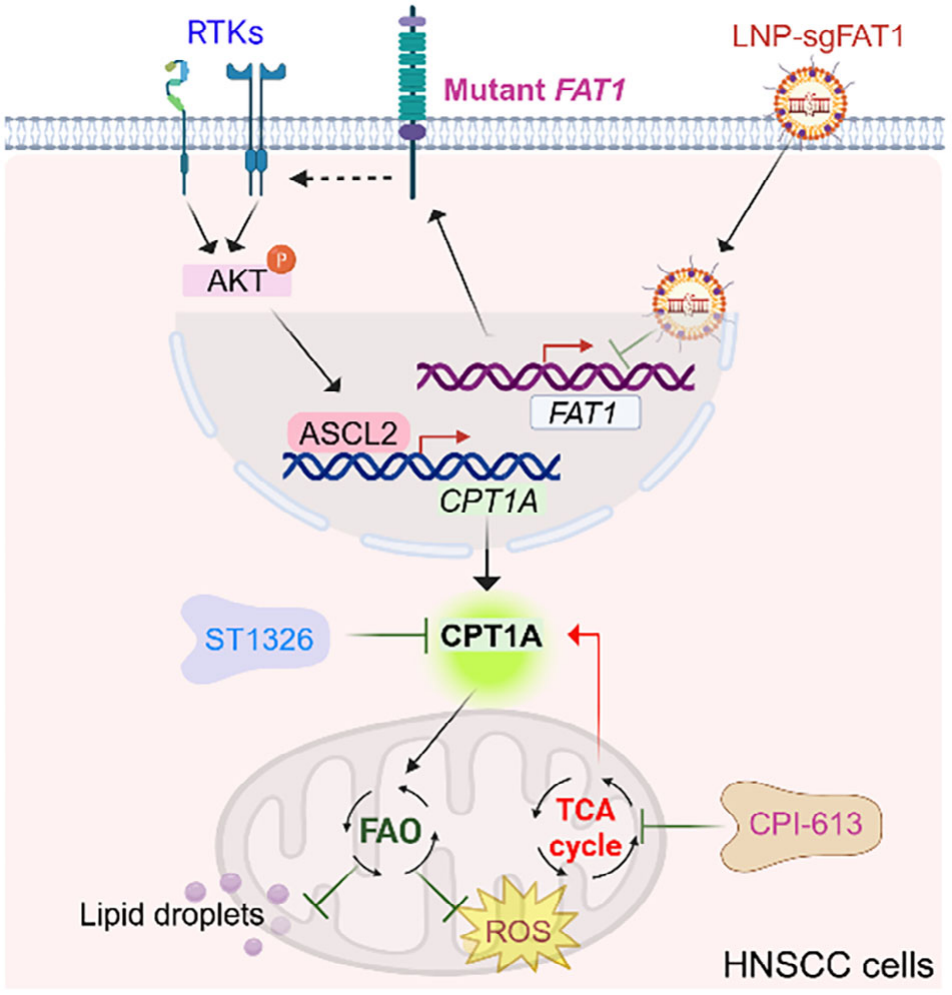
The schematic illustrates the underlying mechanism by which mutant *FAT1* influences treatment efficacy of TCA cycle inhibition in HNSCC cells. CPI‐613 treatment inhibits the TCA cycle while increases CPT1A levels in HNSCC cells harboring mutant *FAT1*. Inhibiting FAT1 using LNP‐sgFAT1 decreases CPT1A expression by suppressing ASCL2, leading to reduced fatty acid oxidation (FAO). This reduction in FAO subsequently results in an increase in lipid droplets and reactive oxygen species (ROS), thereby potentiating the antitumor effects of CPI‐613. Figure created using Biorender (https://biorender.com/).

Collectively, these findings suggest that inhibiting mutant *FAT1* reduces CPT1A levels by downregulating the AKT‐ASCL2 signaling axis, thereby reducing FAO and leading to LDs and ROS accumulation in HNSCC cells (**Figure** **7**). Based on this mechanism, LNP‐sgFAT1 we developed has the potential to suppress CPI‐613‐induced FAO, thereby enhancing its antitumor efficacy (Figure 7).

## 3 Discussion

The role of *FAT1* in both normal and cancer tissues has been extensively investigated since its initial discovery in *Drosophila*.^[^ ^34^, ^35^, ^36^ ^]^ We and other groups have demonstrated that wild‐type *FAT1* serves as a tumor suppressor gene in HNSCC.^[^ ^10^, ^11^ ^]^ The *FAT1* gene is one of the most frequently mutated genes across various cancers, with a particularly high prevalence in HNSCC.^[^ ^37^ ^]^ However, the functions of mutant *FAT1* remain to be elucidated. Our study focused on elucidating the role of mutant *FAT1* in the development and treatment response of HNSCC, as we identified a significant negative prognostic impact of mutant *FAT1* on OS in HPV‐ HNSCC patients using a nomogram model. *FAT1* in SCC1 and TU686 cells harbors multiple point mutations rather than a single mutation,^[^ ^11^, ^38^ ^]^ making it technically unfeasible to introduce the exact set of mutations into HNSCC cells with wild‐type *FAT1* to assess their impact on growth advantage. Furthermore, the absence of hotspot mutations in *FAT1* complicates the identification of specific alterations that may contribute to a potential growth advantage.

Our study further showed strong association of mutant *FAT1* with FAO involving CPT1A. Loss of mutant *FAT1* reduced FAO activity in HNSCC cells via downregulating the expression of *CPT1A*. CPT1A is a key regulator of FAO, a metabolic pathway that plays a critical role in energy production. FAs serve as an important energy source, complementing glucose and glutamine, particularly in conditions where these substrates are limited or when cells require additional metabolic flexibility, such as in cancer cells.^[^ ^15^ ^]^ Mitochondrial FAO is a complex process that relies on more than two dozen enzymes and transport proteins responsible for the activation and transport of FAs into the mitochondria. Among these, carnitine CPT1 serves as the key rate‐limiting enzyme of FAO, playing a critical role in regulating the entry of FAs into the mitochondria for oxidation.^[^ ^15^ ^]^ The CPT1 family of proteins consists of three isoforms: CPT1A, CPT1B, and CPT1C. Among these, CPT1A has been widely studied and is frequently overexpressed in various types of cancers, including HNSCC. This overexpression is often associated with enhanced FAO, which supports cancer cell survival, growth, and metabolic adaptation in nutrient‐scarce or stressful environments.^[^ ^15^ ^]^ The prominence of CPT1A in cancer metabolism also contributes to metastasis of triple negative breast cancer,^[^ ^39^ ^]^ colorectal cancer,^[^ ^40^ ^]^ and ovarian cancers.^[^ ^41^ ^]^ Elevated CPT1A also reduces the sensitivity of myeloma^[^ ^42^ ^]^ and leukemia to anticancer agents.^[^ ^21^ ^]^ In advanced oropharyngeal squamous cell carcinoma (OPSCC), one of the major subsets of HNSCC, overexpression of *CPT1A* was found to be associated with prognosis and response to chemoradiation therapy.^[^ ^43^ ^]^ Our study suggests that mutant *FAT1* may drive CPT1A overexpression by activating the AKT‐ASCL2 signaling axis in HNSCC cells, thereby promoting FAO to meet the energy demands of cancer cells. Further studies are needed to explore how AKT signaling interacts with ASCL2 in the context of mutant *FAT1*.

CPI‐613 is primarily designed to treat pancreatic cancer.^[^ ^17^, ^18^, ^44^ ^]^ One of our mechanistic studies indicates that CPI‐613 exhibits anticancer activity in pancreatic cancer cells by triggering ROS‐associated apoptosis, which is accompanied by increased autophagy and repressed lipid metabolism through activating AMPK‐acetyl‐carboxylase (ACC) signaling.^[^ ^45^ ^]^ Our investigation of this compound has expanded to other types of cancer, including HNSCC, where CPI‐613 has demonstrated dose‐dependent anti‐tumor activity.^[^ ^46^ ^]^ In addition to demonstrating potent anticancer effects, CPI‐613 has exhibited excellent tolerance in clinical trials. It is currently being investigated in combination with various chemotherapeutic agents for the treatment of several cancers, including metastatic pancreatic cancer (NCT03504423), biliary tract cancer (NCT04203160), clear cell sarcoma (NCT04593758), and relapsed/refractory acute myeloid leukemia (NCT03504410). These ongoing clinical trials highlight its potential as a promising therapeutic agent across a range of malignancies. However, the therapeutic potential of CPI‐613 in HNSCC patients or in combination with other therapy has yet to be investigated. Our bioinformatics analysis, leveraging integrated data from TCGA and GDSCs, revealed that mutant *FAT1* is associated with sensitivity to several existing and/or potential anticancer agents, including CPI‐613. In this study, we have demonstrated for the first time that CPI‐613 can induce CPT1A expression, and that either inhibiting CPT1A with its specific inhibitor or reducing CPT1A levels by depleting mutant *FAT1* significantly enhances the antitumor efficacy of CPI‐613. These novel findings suggest that FAT1 may act as a key regulator of CPT1A‐mediated FAO, potentially creating a metabolic bypass that compensates for CPI‐613‐induced suppression of the TCA cycle in HNSCC cells. One possible mechanism involved in CPI‐613‐induced CPT1A upregulation is energy stress activation. CPI‐613 reduces ATP production by impairing the TCA cycle, leading to activation of AMP‐activated protein kinase (AMPK), a key regulator of cellular energy homeostasis and CPT1A.^[^ ^47^, ^48^ ^]^ AMPK activation subsequently upregulates CPT1A to enhance FAO as a compensatory energy source. A second possible mechanism underlying CPI‐613‐induced CPT1A upregulation is acetyl‐CoA depletion. By inhibiting PDH, CPI‐613 reduces acetyl‐CoA availability, restricting substrate input into the TCA cycle, which may trigger compensatory FAO activation, where CPT1A expression is upregulated to increase fatty acid flux into mitochondria. Another potential mechanism is nutrient sensing pathways. CPI‐613‐induced metabolic stress may engage transcription factors such as PPARα or FOXA2, which are known to regulate CPT1A expression in response to metabolic changes,^[^ ^49^, ^50^ ^]^ promoting FAO as an adaptive response. Nevertheless, the precise mechanism underlying CPI‐613‐induced CPT1A upregulation and its relationship with mutant *FAT1* remains to be fully elucidated.

In a recent study, Masarwy et al. developed a CRISPR/Cas9‐LNP system to selectively knockout SOX2 in HNSCC cells.^[^ ^51^ ^]^ This genome‐editing strategy effectively inhibited tumor cell growth in vitro and demonstrated significant therapeutic efficacy in tumor‐bearing mice following intratumoral injection, leading to a 90% increase in survival (>84 days). These findings underscore the potential of the LNP‐mediated gene editing platform as a promising approach for treating aggressive and clinically accessible tumors. In their study, an EGFR antibody was conjugated to the LNP surface to enhance mRNA delivery. In contrast, our research demonstrates that a ligand‐free formulation, based on the polyamine and chalcogen ether lipid 113‐O14O, is highly effective in transfecting HNSCC cells and mediating CRISPR genome editing. Furthermore, our LNP formulation differs from the one developed by Masarwy et al. in terms of both lipid composition (e.g., ionizable lipid and phospholipid) and fabrication methodology.^[^ ^51^ ^]^ The two‐step formulation process is a well‐established approach in our lab to fabricate mRNA/sgRNA/LNP formulations.^[^ ^33^, ^52^ ^]^ While the one‐step formulation process may provide some advantages in certain cases,^[^ ^53^, ^54^ ^]^ we chose the two‐step post‐mixing approach primarily to minimize the potential risk of mRNA degradation during the process of nanoparticle formulation, fabrication and purification. It is important to note that we used an orthotopic tongue tumor model, which more accurately represents the pathophysiology of HNSCC compared to the ectopic subcutaneous HNSCC mouse model. In addition to assessing CRISPR‐Cas9 monotherapy, we also explored the therapeutic effects of combining CRISPR‐Cas9 genome editing with the small‐molecule cancer metabolism inhibitor CPI‐613. This combination therapy demonstrated enhanced antitumor efficacy, outperforming the effects of either treatment individually.

Relative to HPV+ HNSCC, HPV‐ HNSCC is less sensitive to conventional cytotoxic therapy and often develops intrinsic and/or acquired resistance to treatment. Interestingly, the mutation rate of *FAT1* is significantly greater in HPV‐ HNSCC compared to HPV+ HNSCC, underscoring the importance of *FAT1* mutations in the molecular landscape of HNSCC and suggests that FAT1 may serve as a critical biomarker or therapeutic target, particularly in HPV‐ cases. However, a significant clinical challenge lies in achieving targeted depletion of mutant *FAT1* in vivo. In this study, we demonstrated the therapeutic potential of gene‐editing technology using LNPs as a delivery system. We utilized our previously developed chalcogen‐based LNPs to deliver Cas9 mRNA and sgRNAs targeting the *FAT1* gene locus in HPV‐ HNSCC cells, termed LNP‐sgFAT1. The mRNA‐sgRNA/LNP complex exhibited optimal physicochemical properties, including a favorable hydrodynamic size, uniform morphology, excellent mono‐dispersity, and high RNA encapsulation efficiency. These characteristics highlight the potential of this delivery system for efficient and precise gene editing in cancer therapy. In an in vivo study using an orthotopic SCC1 tongue tumor‐bearing mouse model, *FAT1* depletion mediated by LNP‐sgFAT1 led to a reduced tumor growth rate and smaller tumor size. Moreover, the combination of LNP‐sgFAT1 and CPI‐613 demonstrated significantly enhanced antitumor effects compared to monotherapy, as evidenced by a decreased tumor burden and prolonged survival. These studies provide proof‐of‐concept for the rational design of precision medicine in cancer therapy, achieved through the integration of LNP‐enabled CRISPR genome editing and a metabolism inhibitor with a well‐defined mechanism of action.

In summary, this study provides compelling evidence for the regulatory role of mutant *FAT1* in FAO and its influence on sensitivity to the anticancer agent CPI‐613. These findings suggest that targeting mutant *FAT1*, in combination with CPI‐613, could represent a promising new therapeutic strategy for treating HPV‐ HNSCC patients harboring FAT1 mutations. This innovative approach highlights the potential for synergistic therapeutic strategies in targeting cancer‐specific genetic and metabolic vulnerabilities.

## 4 Experimental Section

### Gene Mutation Analysis Using Dataset from TCGA HNSCC Cohort

Gene mutation data were extracted from TCGA HNSCC dataset using ‘TCGAbiolinks’ R package with ‘Simple Nucleotide Variation’ and ‘Masked Somatic Mutation’ based on ‘open’ data. Gene mutation types, including 3’ Flank, 3’ UTR, 5’ Flank, 5’ UTR, Frame Shift Del, Frame Shift Ins, Missense Mutation, Multi Hit, Nonsense Mutation, In Frame Del, In Frame Ins, Splice Site, and Translation Start Site, were analyzed using ‘Mafttools’ R package. The correlation between OS of HNSCC patients with *FAT1* mutations and their clinical characteristics (age, gender, stage, and M/N/T stage) was calculated using ‘survival’, ‘regplot’, and ‘rms’ R package.

### Comparative Assessment of Drug Sensitivity Based on *FAT1* Wide‐Type and Mutation Status Using Combined HNSCC Datasets from TCGA and GDSCs

To investigate the correlation between *FAT1* mutation status and drug sensitivity in HNSCC patients, models were constructed using TCGA gene expression data and GDSC drug sensitivity data and integrated them based on patient IDs. The expression matrix was constructed based on gene symbol, raw gene counts [log2 (counts+1)] and patient ID using Perl software. Patients were divided into two subgroups based on *FAT1* mutation status (wild‐type vs. mutation). Drug sensitivity data were obtained from GDSCs datasets (367 compounds in GDSC1 dataset and 198 compounds in GDSC2 dataset) and the drug sensitivity matrix was constructed based on patient ID and drug name. Clinical drug sensitivity information for HNSCC patients was obtained from the NCI Genomic Data Commons portal. Drug names and sensitivity information were retrieved from the clinical metadata and edited for typographical and spelling errors and to harmonize commercial names and molecular drug names. Differences in drug sensitivity between the two subgroups (wild‐type *FAT1 vs*. mutant *FAT1*) were evaluated using “limma” and “oncoPredict” R package. *p* < 0.05 was considered statistically significant.

### Cell Lines and Cell Culture

Tu686 cells and JHU029 cells were obtained from Dr. Maie St. John at University of California Los Angeles and Dr. David Sidranski at Johns Hopkins University, respectively. HN12 cells were a kind gift from Dr. Andrew Yeudall in 2016 and maintained in this lab. SCC1 cells were obtained courtesy of Dr. Thomas E. Carey at University of Michigan. Cells were cultured in DMEM/F‐12 medium containing 5% fetal bovine serum (Hyclone) at 37 °C in a humidified incubator supplied with 5% CO_2._ All cell lines were not genetically authenticated but were routinely screened for mycoplasma contamination by MycoAlert Mycoplasma Detection Kit (Lonza) and used for experiments before passage 10.

### Reagents, Plasmids, Antibodies, and Standard Assays

CPI‐613 was obtained from Selleckchem (Houston, TX). Malonate, lonidamine and SC79 were purchased from MCE (Monmouth Junction, NJ). ST1326 and DCF were purchased from Sigma‐Aldrich (St. Louis, MO). CRISPR/Cas9 KO plasmid consisting of *FAT1*‐specific 20 nt sgRNA sequence was obtained from Santa Cruz Biotechnology (Dallas, TX). Full‐length flag‐tagged human wild‐type *FAT1* (wtFAT1 O/E) was a gift from Dr. Mingqian Feng at Huazhong Agricultural University. p7056 pHAGE‐P‐CMVt‐N‐HA‐GAW‐CPT1A was a gift from Peter Howley (Addgene plasmid # 100146; http://n2t.net/addgene:100146; RRID: Addgene_100146).^[^ ^55^ ^]^ BII‐ChiNW‐ASCL2 was a gift from Blair Madison (Addgene plasmid # 133384; http://n2t.net/addgene:133384; RRID: Addgene_133384).^[^ ^56^ ^]^ Antibodies specific for the following proteins were used for Western blot: FAT1 (Abcam; Cat# Ab241372), CPTA1 (Proteintech; Cat# 15184‐1‐AP), ASCL2 (Proteintech; Cat# 21368‐1‐AP), β‐Actin (Sigma Aldrich, Cat# A5316), AKT (Cell Signaling Technology, Cat# 4691), p‐AKT (Cell Signaling Technology, Cat# 4060), Ki67 (Sigma Aldrich, Cat# SAB5600249). Secondary antibodies were purchased from Invitrogen (Carlsbad, CA). Cell viability, colony formation and Western blot assays were carried out as previously described.^[^ ^57^, ^58^ ^]^

### Cas9 mRNA and sgRNAs

SpCas9 mRNA was purchased from TriLink Biotechnologies (San Diego, CA). A scrambled sgRNA (sgNC) and *FAT1*‐targeted sgRNAs (sgFAT1) with chemical modification (2’‐O‐methyl at three first and last bases; 3’‐phosphorothioate bonds between first three and last two bases) were purchased from Synthego (Redwood City, CA). Target sequences were 5’‐AGCGCCTACCATGACCGTGA‐3’ for sgFAT1‐1 and 5’‐TATCGCAAGAGTCAGCGCCA‐3’ for sgFAT1‐2. Unless otherwise specified, sgFAT1 refers to sgFAT1‐1. To quantify CRISPR editing outcomes for sgFAT1, genomic DNA was extracted using the QIAamp DNA Micro Kit (QIAGEN, Germantown, MD). PCR amplification of the *FAT1* locus was performed with the UltraRun LongRange PCR Kit (QIAGEN, Germantown, MD) using specific primers (Forward: CTGCAACACAACTGAGCCAA; Reverse: ACAGCGCCAGAAACAGAAAT). Amplified DNA was purified with the QIAquick Gel Extraction Kit (QIAGEN, Germantown, MD), followed by sequencing conducted at Genewiz Inc. (South Plainfield, NJ). Sequence data was processed using the Synthego ICE platform to assess insertion‐deletion mutations and gene knockout efficiency.

### ChIP‐qPCR Analysis

ChIP assay was carried out using a ChIP Assay Kit (ThermoFisher Scientific), following the previously established procedure.^[^ ^59^ ^]^ Soluble chromatin was immunoprecipitated using an anti‐ASCL2 antibody at a 1:100 dilution, combined with 20 µl of protein A/G beads, and incubated overnight at 4 °C. The immunoprecipitates were washed 3 times and eluted from the beads with elution buffer, and reverse cross‐linked with 0.5m NaCl for 4 h at 65 °C. Cross‐linked chromatin was treated with RNAse A for 1 h at 37 °C and phenol‐extracted, and the eluate was run on an agarose gel to visualize specific DNA‐protein complexes. Quantitative analysis of the relative enrichment of specific DNA sequences was performed using the StepOne Plus Real‐Time PCR System (Applied Biosystems). Primers used for this study are: 5’‐GCTCTCAGTTCTGCAGGCTA‐3’ (forward) and 5’‐CCTTTGCCTTCCACCATGAG‐3’ (reverse).

### Specific Transcription Factor‐DNA Interaction

JASPAR (http://jaspar.genereg.net) was utilized to predict the potential location of the ACSL2 binding site on the *CPT1A* gene promoter. The dCas9 vector p‐LV‐TRE3G‐dCas9‐DsRed‐Zeo with an HA tag was a kind gift from Dr. S. Ali Shariati at UC Santa Cruz,^[^ ^16^ ^]^ which was designed to identify specific transcription factor‐DNA interactions. The sgRNA targeting the *CPT1A* gene promoter (sgTarget, 5’‐AAGCAUGGAGGCAUCUGCUUC‐3’), designed to fully encompass the ASCL2 binding site based on available NGG PAM sites nearby, was co‐transfected with p‐LV‐TRE3G‐dCas9‐DsRed‐Zeo into HNSCC cells, following the CRISPRd approach described in a previous study.^[^ ^16^ ^]^ An sgRNA targeting the *GFP* gene (shGFP) was used as a negative control to assess the specific effects of sgTarget. All sgRNAs used in this study were synthesized by Synthego (Redwood City, CA).

### Lipid Synthesis and Fabrication of LNP Formulations

Amine‐containing ionizable lipid molecules, 113‐O14O, 306‐O10S, and 113‐O12O were synthesized and characterized following the previously reported procedures.^[^ ^22^, ^60^ ^]^ 113‐O14O, 306‐O10S, or 113‐O12O was dissolved in ethanol. Cholesterol (Sigma‐Aldrich), DOPE (1,2‐dioleoyl‐sn‐glycero‐3‐phosphoethanolamine, Avanti Polar Lipids), and DMG‐PEG2k (1,2‐dimyristoyl‐rac‐glycero‐3‐methoxypolyethylene glycol‐2000], Avanti Polar Lipids) were added at a 16/4/2/1 weight ratio (ionizable lipid/cholesterol/DOPE/DMG‐PEG2k). The molar ratios for 113‐O14O‐, 113‐O12O‐, and 306‐O10S‐based LNPs are ≈50/38.5/10/1.5 (113‐O14O (MW = 1199)/cholesterol/DOPE/DMG‐PEG2k), 52/37/10/1.4 (113‐O12O (MW = 1087)/cholesterol/DOPE/DMG‐PEG2k), and 53/36/9.5/1.4 (306‐O10S (MW = 1067)/cholesterol/DOPE/DMG‐PEG2k), respectively. The ethanol solution was mixed with sodium acetate buffer (25 mm, pH 5.2) through a T‐junction (ethanol phase/aqueous phase = 1/3, volume ratio). After dialysis (MWCO 3.5 kDa, Slide‐A‐Lyzer, ThermoFisher Scientific), purified LNPs were mixed with Cas9 mRNA and sgFAT1 or sgNC following a two‐step formulation procedure (LNP/mRNA/sgRNA = 16/1/1, weight ratio), and the complex was incubated for 30 min at room temperature before use.

### Characterization of LNP Formulations

Average hydrodynamic diameter (<*D*_h_>) and PDI of LNP‐sgFAT1 were measured by Zeta‐PALS particle size analyzer (Brookhaven Instruments). RNA encapsulation efficiency was measured using Quant‐it RiboGreen RNA Assay Kit (Invitrogen). CryoEM images were taken on a cryo transmission electron microscope (JEOL JEM‐2100).

### Tumorsphere Formation Assays

Dissociated single HNSCC cells with or without *FAT1* KO were plated on Corning Costar 6‐well ultra‐low attachment plates (cat# 3471, In Vitro Technologies) at a density of 1 × 10^4^ cells per mL and grown in StemXVivo Serum‐free Tumorsphere media (cat# CCM012, R&D Systems, In Vitro Technologies). Progress was monitored daily under an inverted microscope and fresh media was added every 4 d. After a 2‐week incubation, the number of tumorspheres at least 50 µm in size was counted using a Zeiss microscope.

### RNA‐seq and Bioinformatics Analysis

Total RNA was extracted from *FAT1* KO and parental SCC1 cells using TRIzol reagent (Invitrogen, Carlsbad, CA) according to the manufacturer's protocol. The purified RNA samples were sent to Novogene Corporation (Sacramento, CA) for library construction and sequencing using the Illumina HiSeq 2000 platform to obtain expression libraries of 50‐nt read length. Independent duplicate cultures were sampled to avoid random error. DEGs were identified using the DESeq R package (version 4.1.2.) functions estimateSizeFactors and nbinomTest. *p* < 0.05 and fold change (FC) > 2 or FC < 0.5 were set as the threshold for significantly differential expression. A hierarchical Bayesian model was applied to test differential expression.^[^ ^61^ ^]^ Volcano plot was generated based on the log2 *FC* and the ‐log10 *False_Discovery_Rate (FDR)* of each gene. The KEGG pathway gene set enrichment analysis was performed based on ‐log10 *FDR* and the sign of log2 *FC* of each gene by function gseKEGG in the Bioconductor package clusterProfiler. 0.05 was set as the significance level, and the significant pathways were divided into activated and suppressed groups by assessing whether the corresponding NES was greater than zero or not. The Bioconductor package enrichplot was applied in visualizing the GSEA results. Dot plot was generated based on the enrichment score (adjusted p‐value) and gene count (number of core genes) and ratio (count divided by set size of the pathway) as height and color using function dotplot. Gene network plot, upset plot, and heatmap were generated by the number of core genes (size) of pathways and significance level of pathways and genes using function cnetplot upsetplot, and heatplot, respectively. To draw a ridge plot, the ‐log10 *FDR* for core genes for KEGG GSEA enriched categories and the P‐value of each pathway were used in function ridgeplot. Enrichment score plot was generated by enrichment score using the function gseaplot2.

### Phospho‐RTK Profiling

The Proteome Profiler Human Phospho‐RTK Array Kit (R&D Systems, MN) was used for phospho‐RTK profiling. In brief, 500µg of freshly isolated protein from FAT1 KO or parental SCC1 cells was diluted and incubated overnight with nitrocellulose membranes containing duplicate spots for 42 anti‐RTK and control antibodies. Bound phospho‐RTKs were detected using a pan anti‐phosphotyrosine antibody conjugated to horseradish peroxidase, followed by visualization with ECL reagents from Bio‐Rad (Hercules, CA).

### FAO Activity Assay

FAO enzyme activity was measured using a FAO Assay Kit (Biomedical Research Service Center, E‐141) according to the manufacturer's instructions. All samples were harvested with 1×Cell Lysis Solution. Protein concentration of the samples was determined by a BCA assay and normalized to 1 mg ml^−1^. Twenty microliters of each sample was added in triplicate to a 96‐well plate on ice. Immediately, 50 µl control solution was added to one set of wells and 50 µl of reaction solution to the other set of wells. The contents were mixed by gentle swirling for 10 s. The plate was covered and incubated in a 37 °C humidified incubator for 30 min. Optical density was read at of 492 nm (OD 492) using a microplate reader (Bio‐Rad). The control well reading was subtracted from the reaction well reading for each sample and the subtracted OD represents the FAO activity of the sample.^[^ ^62^ ^]^

### Measurement of FAO‐Associated OCR

Approximately 4 × 10^4^ *FAT1* KO or parental SCC1 cells, treated with or without 50 µm CPI‐613 for 24 h, were seeded directly onto Seahorse XFPS plates (Agilent Technologies) per well. OCR was acquired in cells using the Seahorse XF Long Chain Fatty Acid Oxidation Stress Test Kit (Agilent Technologies) by sequential treatment with DMEM medium or 4 µm etomoxir (Eto), 1.5 µm oligomycin, 1 µm FCCP, and 0.5 µm antimycin A/rotenone and analyzed using a Seahorse XFe24 flux bioanalyzer (Agilent Technologies).

### LD Staining

For fluorescence imaging, live cells were stained with BODIPY 493/503 (5 µg ml^−1^ in PBS, protected from light) (Invitrogen) for 30 min. Cells were then washed three times with PBS and fixed with 4% paraformaldehyde, followed by a 10 min staining with DAPI (Invitrogen). Fluorescence imaging was conducted using a ZEISS LSM710 Confocal laser scanning microscope. For flow cytometry, cells stained with 0.5µg mL^−1^ Nile Red (Invitrogen) were collected with a cell scraper and filtered through a 0.40 µm membrane filter, and the percentage of fluorescent cells in the population and the fluorescence intensity were measured using a flow cytometer (BD FACSAria II).

### Animal Studies

Six‐week‐old male NOD.Cg‐*Prkdcscid Il2rgtm1Wjl/SzJ* (NSG) mice were purchased from the Jackson Laboratory (Bar Harbor, ME). All animal experiments were approved by the Institutional Animal Care and Use Committee (IACUC) of Emory University (PROTO202100085). To determine the effect of *FAT1* KO on tumor growth, 1 × 10^6^ *FAT1* KO or parental SCC1 cells were suspended in 100µL of PBS/Matrigel (3:1) and injected into the right flank of NSG mice. To assess the therapeutic efficacy of LNP‐sgFAT1, an orthotopic xenograft tongue tumor model was established by injection of 1 × 10^5^ SCC1 cells into the anterior portion of the tongue of NSG mice. When the tongue tumors were established (10 days after cancer cell inoculation), mice were randomized to receive treatments with 5 mg kg^−1^ LNP‐sgFAT1 or the control blank LNPs. To assess the effect of *FAT1* KO on the sensitivity to CPI‐613, NSG mice bearing orthotopic SCC1 tumors with or without *FAT1* KO were treated with vehicle or CPI‐613. To evaluate the therapeutic efficacy of the combination of LNP‐sgFAT1 and CPI‐613, NSG mice bearing SCC1 tumors received vehicle (control blank LNPs in combination with PBS), LNP‐sgFAT1 and CPI‐613, alone or in combination. LNP‐sgFAT1 at a dose of 5 mg kg^−1^ was administered by intratumoral injection three times with an interval of 3 days. CPI‐613 at a dose of 50 mg kg^−1^ was administered by intraperitoneal (*i.p*.) injection every day for 10 days. Tumor dimensions were serially measured with electronic calipers, and tumor volume was calculated by the formula of V = length × width^2^ × 1/2. Afterward, mice were sacrificed on Day 21, and tumors and major organs (including the heart, intestine, kidney, liver, lung, and spleen) were excised for histopathological and immunohistochemistry (IHC) analysis. BMI of animals was quantified at the end of the study by using the formula of BMI = mass (g)/length (cm)^2^. Length was determined by measuring the animal from the nose to the anus. Another cohort of mice with the above treatments was used to calculate the survival curve.

### IHC and IF

Tissue sections were deparaffinized with xylene, rehydrated through a graded alcohol series, and incubated in 3% hydrogen peroxide. Sections were placed in 10 mm sodium citrate buffer (pH 6.0) at sub‐boiling temperatures for 10 min and incubated with 10% normal goat serum, followed by incubation with anti‐Ki67 antibody. Immunoreactivity was visualized using the DAB Detection kit (Vector Laboratories, Burlingame, CA) and counterstained with hematoxylin. Slides were then dehydrated, mounted, and scanned using the Olympus Nanozoomer whole slide scanner (Olympus, Center Valley, PA). For IF, after antigen retrieval and blocking, tumor tissue sections were incubated with anti‐CPT1A antibody overnight at 4 °C, followed by incubation with fluorescence‐conjugated secondary antibody in the dark for 1 h. Slides were mounted with Vectashield mounting medium (Vector Laboratories) containing the DAPI nuclear stain before examination under an inverted fluorescence microscope (BZ‐X710 All‐in‐one, Keyence).

### Apoptosis Analysis

For in vitro analysis, apoptosis was determined by flow cytometry using the Annexin V: PE Apoptosis Detection Kit (Invitrogen, Carlsbad, CA) with 7‐AAD. For in vivo analysis, apoptosis was determined by staining for DNA fragmentation using In Situ Cell Death Detection Kit, TMR red (Roche). The average number of TUNEL‐positive cells in each section in different treatments were counted in twenty randomly selected fields of view using a fluorescence microscope.

### Statistical Analysis

Statistical analysis was performed with statistical software GraphPad Prism 9 (San Diego, CA). Each figure legend denotes the statistical test used. All central tendencies indicate the mean, and all error bars indicate SD. Survival curves were drawn using the Kaplan‐Meier method, and the differences between the two curves were compared with the log‐rank Mantel‐Cox test. Statistical significance was determined using Student's *t*‐test or two‐way ANOVA. Differences were considered statistically significant when *p* < 0.05.

## Conflict of Interest

YT has previously received funds for research contracts from Cornerstone Pharmaceuticals. NFS reports compensated and uncompensated advisory roles with: Astra Zeneca, Eisai Medical, Exelixis, Merck, Merck EMD Serono, Pfizer, Kura, Vaccinex, CUE, BionTech, GSK, TOSK, Seagen, Flamingo, Infinity, Inovio, Aveo, Medscape, Onclive, Uptodate, BMS, Cornerstone, Celldex, Surface Oncology, Astex, Imugene, Faron Pharmaceutical, Coherus, Adagene, Fulgent Springer, Nanobiotix, and Taiho; funding from: Exelixis, BMS. The other authors declare no conflict of interest.

## Supporting information



Supporting Information

## Data Availability

The data that support the findings of this study are available from the corresponding author upon reasonable request.
