# Supplementary material for: Inhibiting FAT1 Blocks Metabolic Bypass to Enhance Antitumor Efficacy of TCA Cycle Inhibition through Suppressing CPT1A‐Dependent Fatty Acid Oxidation
Source: Adv Sci (Weinh). 2025 May 23;12(30):e02146. doi: 10.1002/advs.202502146 (PMC12376706; doi:10.1002/advs.202502146)
Supplement: Supplementary file 1 — Supporting Information [file ADVS-12-e02146-s001.pdf]

ADVANCED SCIENCE
Open Access

Supporting Information

for *Adv. Sci.*, DOI 10.1002/advs.202502146

Inhibiting FAT1 Blocks Metabolic Bypass to Enhance Antitumor Efficacy of TCA Cycle Inhibition through Suppressing CPT1A-Dependent Fatty Acid Oxidation

Fanghui Chen, Jianqiang Yang, David O. Popoola, Fan Yang, Yajie Liu, Dongsheng Wang, Zhaohui S. Qin, Zhengjia Chen, Nabil F. Saba, Zhuo G. Chen, Yamin Li and Yong Teng**

Supplemental Information for

Inhibiting FAT1 blocks metabolic bypass to enhance antitumor efficacy of TCA cycle inhibition through suppressing CPT1A-dependent fatty acid oxidation

Chen *et al*

Correspondence: Yong Teng, yong.teng@emory.edu

This file includes:

Supplementary Figures (Figure S1-Figure S15) and Figure Legends

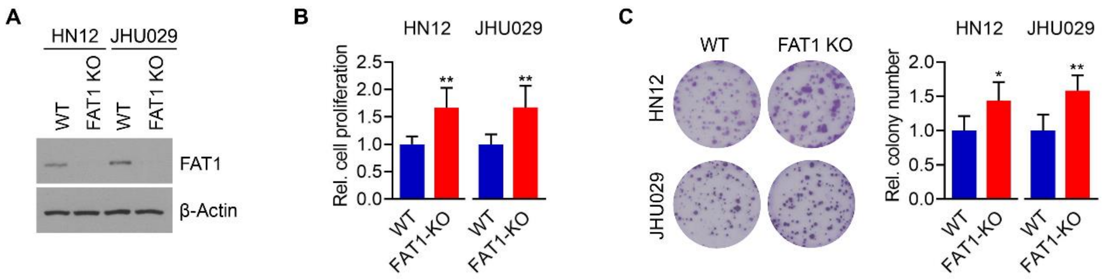

Figure S1. Loss of wild-type *FAT1* promotes HNSCC cell proliferation and clonogenicity. (A) *FAT1* KO efficiency in HN12 and JHU029 cells determined by Western blot. (B, C) Effect of *FAT1* KO on cell proliferation (for 3 days), colony formation (for 14 days) in HN12 and JHU029 cells. Bars express mean ± SD. Statistical analyses were conducted using unpaired two-tailed Student's t-test.

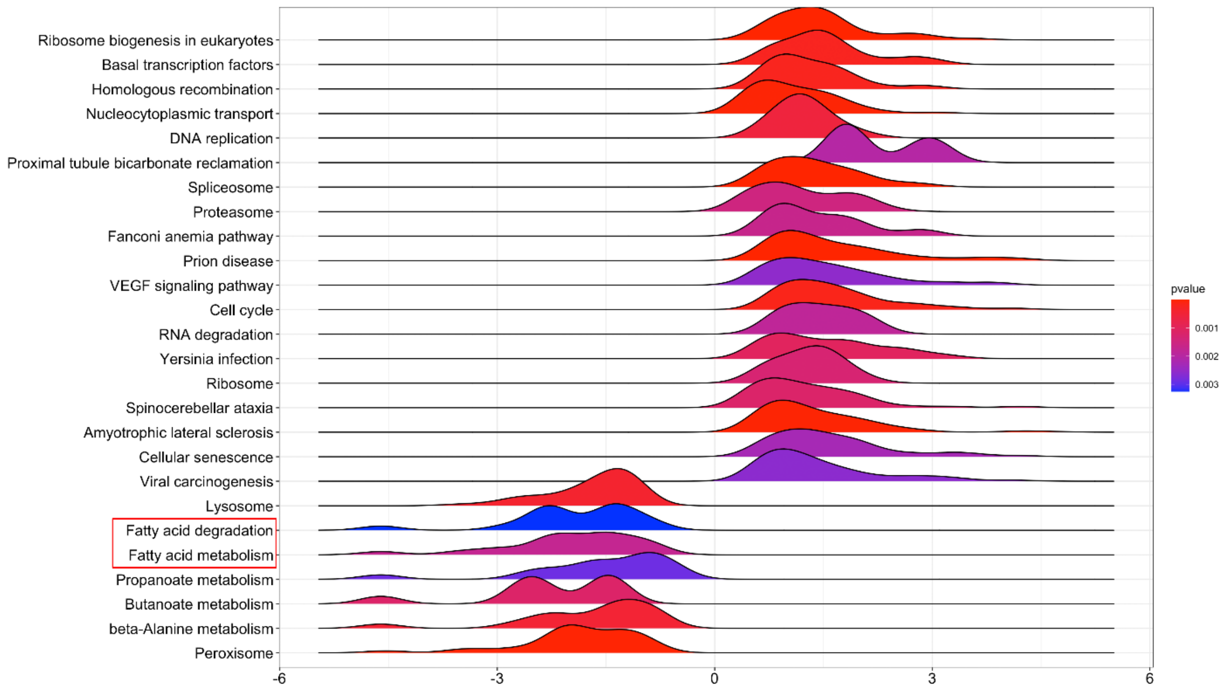

Figure S2. A ridge plot of DEGs in *FAT1* KO *vs.* parental SCC1 cells. The signaling pathways associated with FAM are outlined in red.

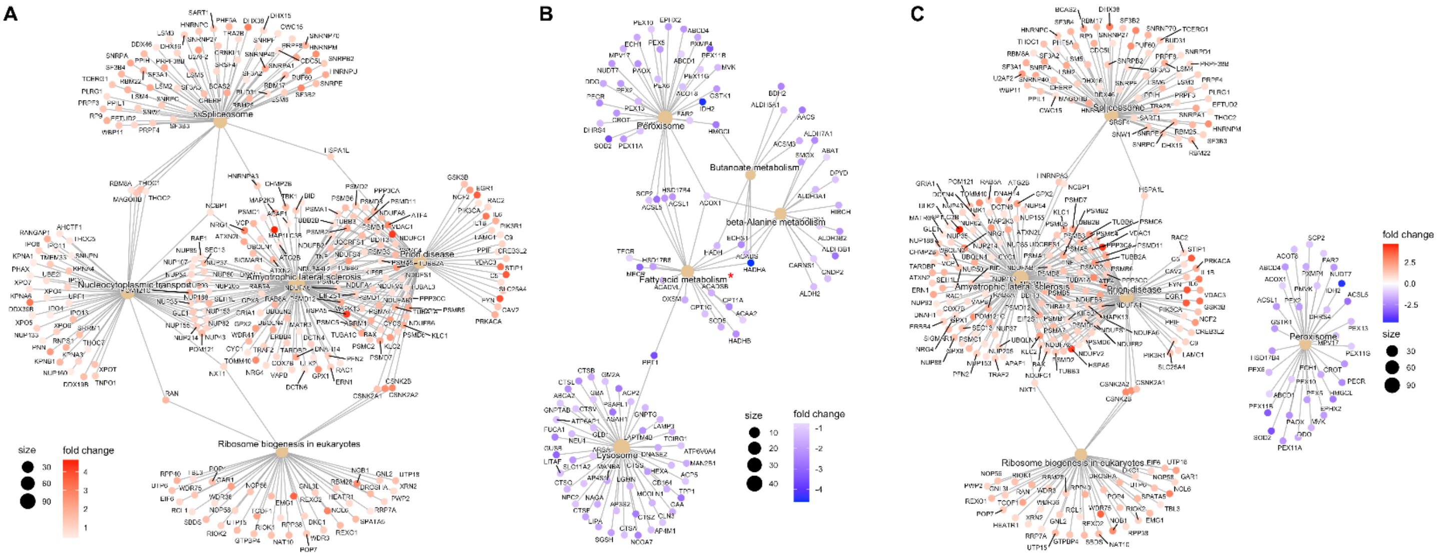

Figure S3. Gene network plots of differentially expressed genes (DEGs) in *FAT1* KO *vs.* parental SCC1 cells. (A) Gene network plots of upregulated DEGs in *FAT1* KO *vs.* parental SCC1 cells. (B) Gene network plots of downregulated DEGs in *FAT1* KO *vs.* parental SCC1 cell. The signaling network associated with FAM is marked with a red star. (C) Gene network plots of all DEGs in *FAT1* KO *vs.* parental SCC1 cells.

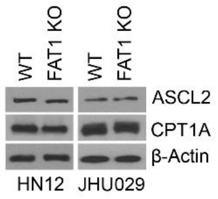

Figure S4. Loss of wild-type *FAT1* does not affect ASCL2 and CPT1A levels. Effect of *FAT1* KO on ASCL2 and CPT1A levels in HN12 and JHU029 was determined by Western blot.

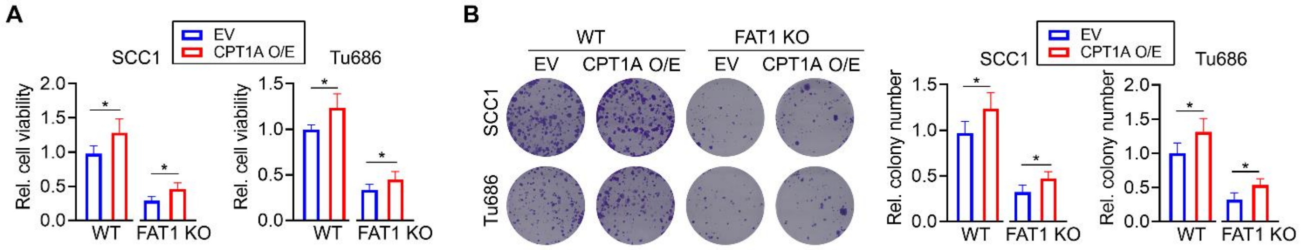

Figure S5. Restoration of *CPT1A* expression in *FAT1* KO cells reverses the suppression of cell proliferation and clonogenicity mediated by *FAT1* KO. Effect of *CPT1A* overexpression (CPT1A O/E) on cell proliferation (A) and colony formation (B) of *FAT1* KO and parental SCC1 and Tu686 cells. Bars express mean ± SD. Statistical analyses were conducted using unpaired two-tailed Student's t-test. *$p<0.05$.

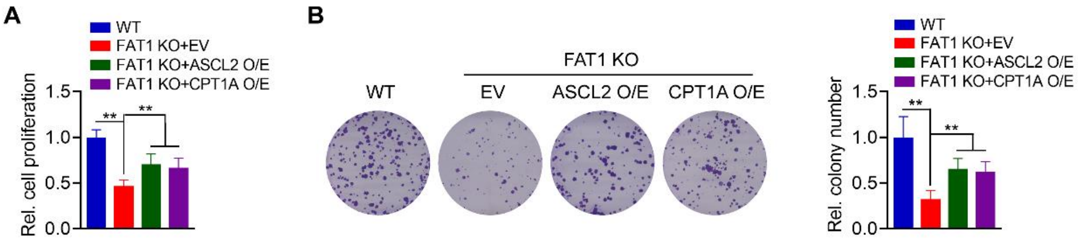

Figure S6. Restoring *ASCL2* or *CPT1A* expression in *FAT1* KO SCC1 cells reverses the inhibition of cell proliferation and clonogenicity mediated by *FAT1* KO. Cell proliferation (A) and colony formation (B) in the indicated treatment groups. EV, empty vector; ASCL2 O/E, *ASCL2* overexpression; CPT1A O/E, *CPT1A* overexpression. Bars express mean ± SD. Statistical analyses were conducted using unpaired two-tailed Student's *t*-test. **p*<0.05; ***p*<0.01.

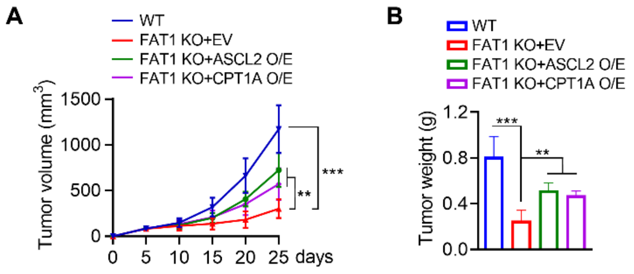

Figure S7. Restoring *ASCL2* or *CPT1A* expression in *FAT1* KO SCC1 cells reverses the suppression of tumor growth mediated by *FAT1* KO in an orthotopic mouse model. Tumor growth curve (A) and tumor weight in the indicated treatment groups. EV, empty vector; ASCL2 O/E, *ASCL2* overexpression; CPT1A O/E, *CPT1A* overexpression. Bars express mean ± SD. Statistical analyses were conducted using unpaired two-tailed Student's *t*-test. *p<0.05; **p<0.01, ***p<0.001.

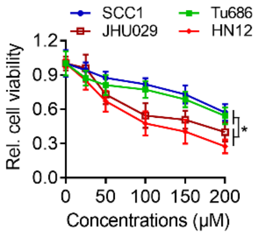

Figure S8. HNSCC cells harboring mutant *FAT1* are less sensitive to CPI-613 compared to those harboring wild-type *FAT1*. SCC1, Tu686, HN12, and JHU029 cells were treated with different concentrations of CPI-613 for 72 hours before cell viability analysis. Bars express mean ± SD. Statistical analyses were conducted using unpaired two-tailed Student's *t*-test. *p<0.05.

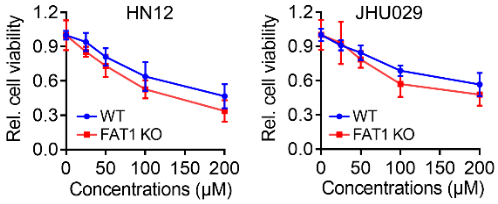

Figure S9. Loss of wild-type *FAT1* in HNSCC cells doesn't change the sensitivity to CPI-613. *FAT1* KO and parental HN12 and JHU029 cells were treated with different concentrations of CPI-613 for 72 hours before cell viability analysis. Bars express mean ± SD. Statistical analyses were conducted using unpaired two-tailed Student's t-test.

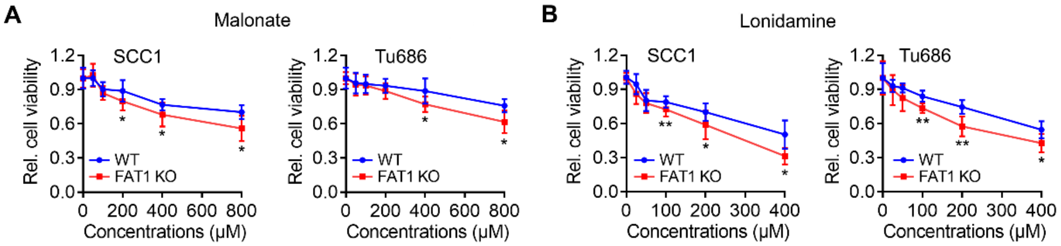

Figure S10. Loss of mutant *FAT1* enhances sensitivity of HNSCC cells to malonate (A) and lonidamine (B). FAT1 KO and parental SCC1 and Tu686 cells were treated with different concentrations of malonate or lonidamine for 72 hours before cell viability analysis. Bars express mean ± SD. Statistical analyses were conducted using unpaired two-tailed Student's *t*-test. *p<0.05; **p<0.01.

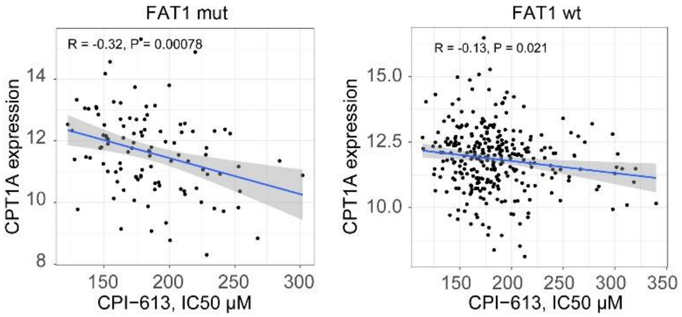

Figure S11. Correlation between expression of *CPT1A* and CPI-613 drug sensitivity (IC50) in HNSCC carrying *FAT1* mutations (mut) *vs.* wild-type (wt).

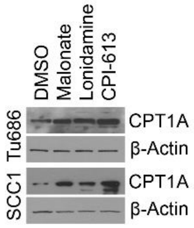

Figure S12. The TCA cycle inhibitors malonate, lonidamine and CPI-613 have the potential to upregulate CPT1A in HNSCC cells with mutant *FAT1*. SCC1 and Tu686 cells were treated with 100μM malonate, lonidamine or CPI-613 for 24 hours, and CPT1A levels were determined by Western blot.

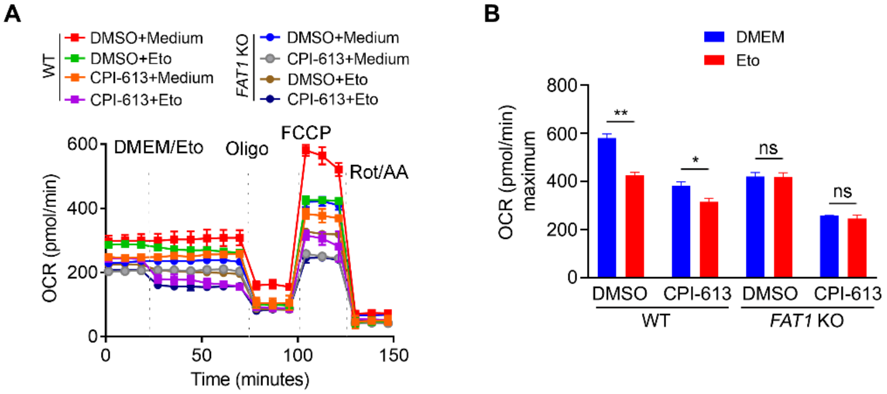

Figure S13. Effect of CPI-613 on FAO dependency in *FAT1* KO and parental SCC1 cells. (A) OCR profiles of SCC1 cells under different conditions measured by a Seahorse XFe24 flux bioanalyzer. In this analysis, *FAT1* KO and parental SCC1 cells were treated with 50 µM CPI-613 for 24 hours. OCR was acquired in cells using the Seahorse XF Long Chain Fatty Acid Oxidation Stress Test Kit on the XFe24 flux bioanalyzer. (B) Changes in OCRs associated with maximal respiratory status under different conditions. Bars express mean ± SD. Statistical analyses were conducted using unpaired two-tailed Student's *t*-test. *p<0.05; **p<0.01.

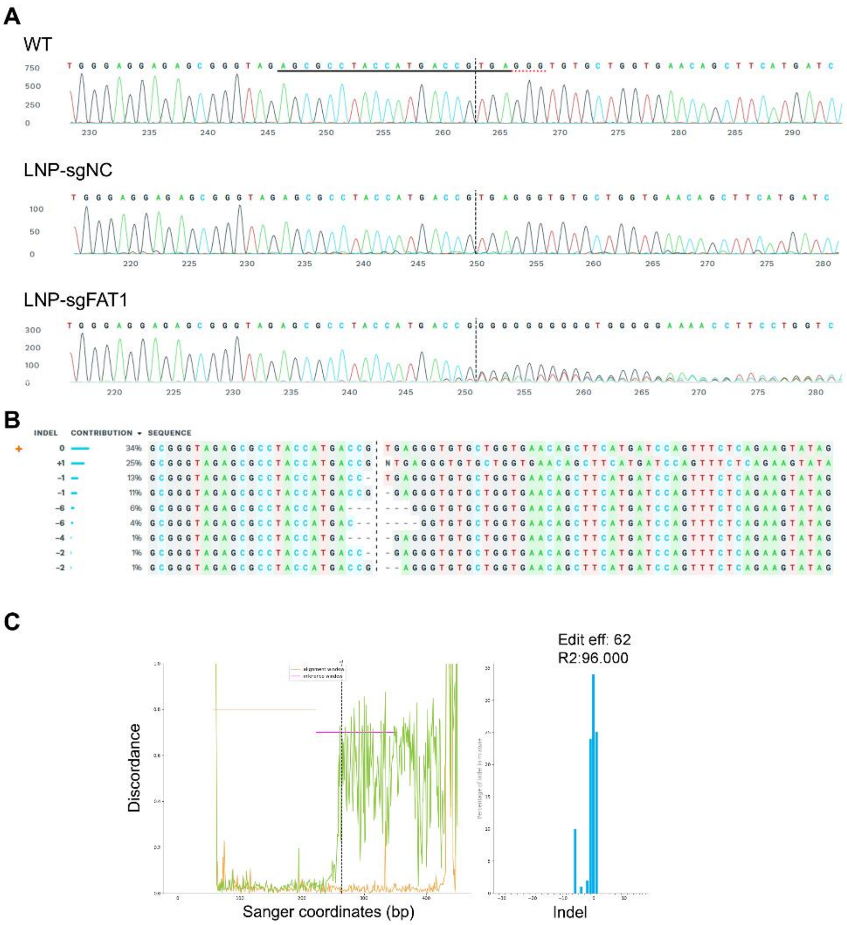

Figure S14. Characterization and genome editing efficiency of LNP-sgFAT1. (A) Sanger sequencing analysis of DNA extracted from SCC1 cells treated with or without LNP-sgFAT1 or LNP-sgNC (scrambled sgRNA control), with the cutting site indicated by a vertical dotted line. (B) Indel contribution in the edited population and their edited proportions in SCC1 cells treated with LNP-sgFAT1. The cutting site was marked by a vertical dotted line and wild-type signal indicated by an orange "+" symbol. (C) Discordance and Indel distribution plots showing the genomic coordinates and editing effects of LNP-sgFAT1.

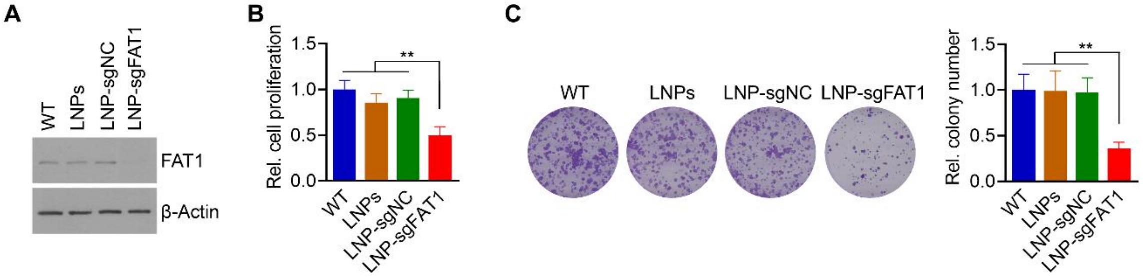

Figure S15. Specific *FAT1*-targeting effect of LNP-sgFAT1. (A) Effect of blank LNPs, LNP-sgNC, and LNP-sgFAT1 on *FAT1* KO in SCC1 cells determined by Western blot. (B, C) Effect of blank LNPs, LNP-sgNC, and LNP-sgFAT1 on cell proliferation (for 3 days) and colony formation (for 14 days). Bars express mean ± SD. Statistical analyses were conducted using unpaired two-tailed Student's *t*-test. *p<0.05; **p<0.01.
